# A change point-based analysis procedure for improving the success rate of decision-making in clinical trials with delayed treatment effects

**DOI:** 10.3389/fphar.2023.1186456

**Published:** 2023-09-11

**Authors:** Long-Shen Xie, Hui Lu

**Affiliations:** Department of Bioinformatics and Biostatistics, School of Life Sciences and Biotechnology, SJTU-Yale Joint Center for Biostatistics and Data Science, Shanghai Jiao Tong University, Shanghai, China

**Keywords:** tumor immunotherapy, delayed treatment effect, change point, interim analysis, decision-making

## Abstract

A delayed treatment effect is a commonly observed phenomenon in tumor immunotherapy clinical trials. It can cause a loss of statistical power and complicate the interpretation of the analytical findings. This phenomenon also poses challenges for interim analysis in the context of phase II/III seamless design or group sequential design. It shows potential to lead researchers to make incorrect go/no-go decisions. Despite its significance, rare research has explored the impact of delayed treatment effects on the decision success rate of the interim analysis and the methods to compensate for this loss. In this study, we propose an analysis procedure based on change points for improving the decision success rate at the interim analysis in the presence of delayed treatment effects. This procedure primarily involves three steps: I. detecting and testing the number and locations of change points; II. estimating treatment efficacy; and III. making go/no-go decisions. Simulation results demonstrate that when there is a delayed treatment effect with a single change point, using the proposed analysis procedure significantly improves the decision success rate while controlling the type I error rate. Moreover, the proposed method exhibits very little disparity compared to the unadjusted method when the proportional hazards assumption holds. Therefore, the proposed analysis procedure provides a feasible approach for decision-making at the interim analysis when delayed treatment effects are present.

## 1 Introduction

**FIGURE 1:**
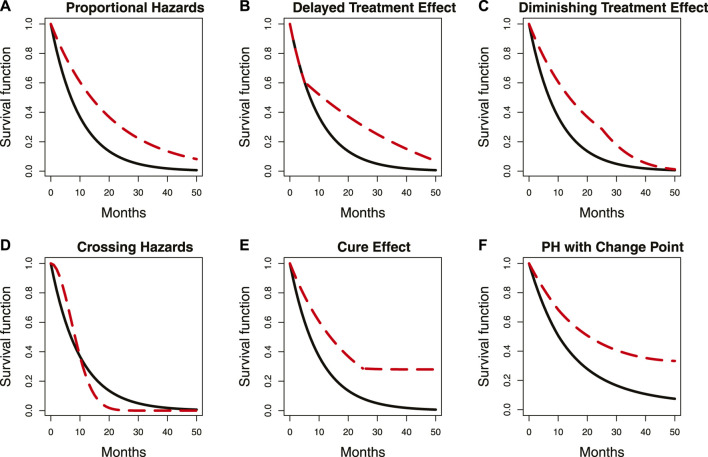
Summary of some common proportional and non-proportional hazard examples. Panels **(A–F)** represent the scenarios described by the captions in each panel. The location of a change point in the survival curve with delayed treatment effects is indicated by the circle. The red dashed line represents survival curves with change points, while the solid black line represents those without change points. PH denotes proportional hazards.

**FIGURE 2:**
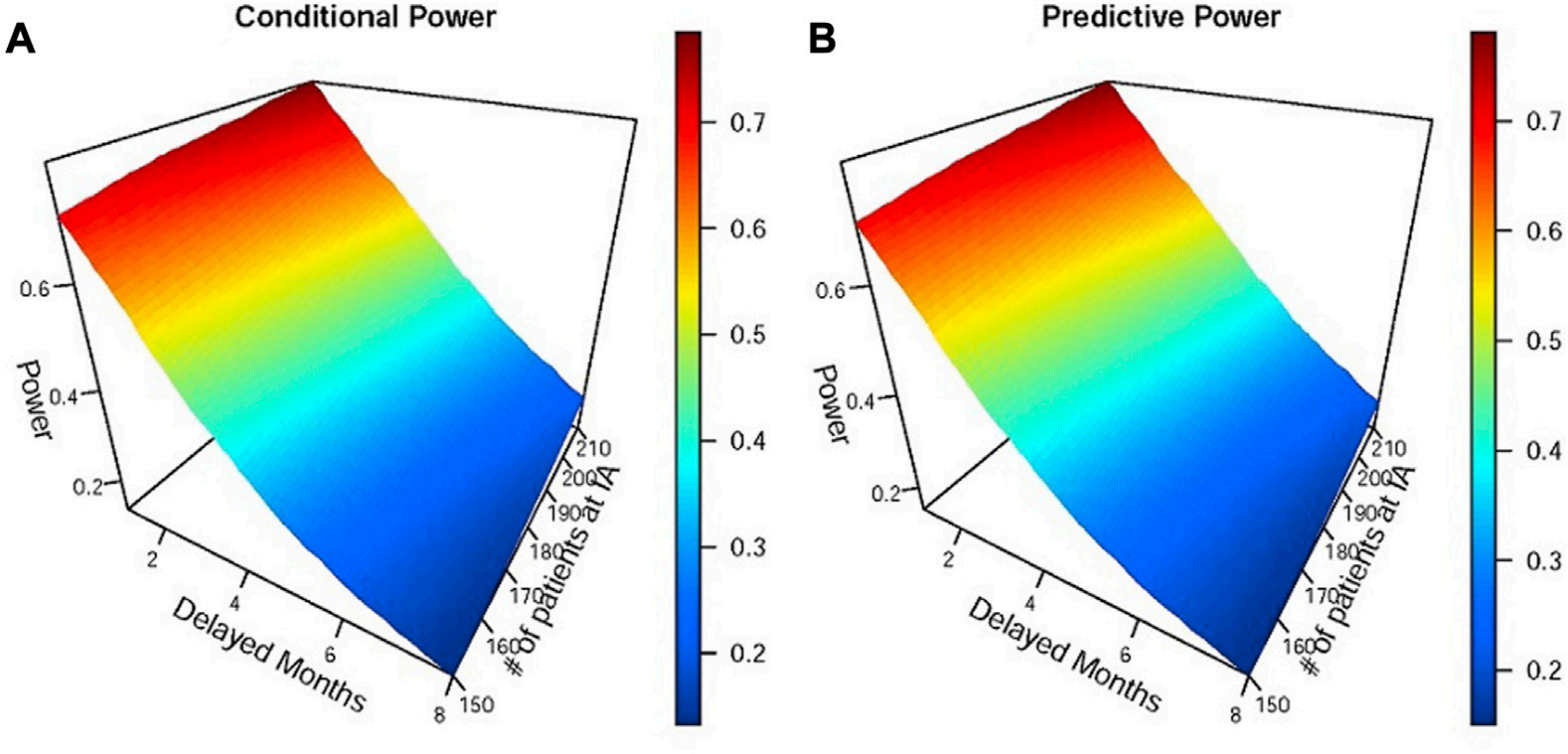
Simulations of the impact of different delayed months on conditional and predictive power at the interim analysis of the group sequential design. Panels **(A)** and **(B)** represent conditional and predictive power, respectively. IA denotes interim analysis. The efficacy parameters used in simulations were as follows: the median OS of the immunotherapy group was 8 and 12 months before and after the delayed effect, and the median OS of the control group was 8 months.

Tumor immunotherapy research has emerged as a prominent focus in clinical drug development (Melero et al., 2015; Farkona et al., 2016) and is one of the most promising areas in the current anti-cancer drug research and development pipeline. The mechanism of action of tumor immunotherapy involves the following key steps (Freeman et al., 2000; Ribas and Wolchok, 2018): i) inhibiting the activity of immune checkpoints; ii) releasing the immune “brake” in the tumor microenvironment; and iii) activating anti-cancer immune responses. Consequently, immunotherapy needs time to activate immune responses, which results in a typical issue when designing a tumor immunotherapy trial, namely, delayed treatment effects (Hodi et al., 2010; Chen, 2013; Larkin et al., 2015; Mick and Chen, 2015; Kudo, 2019; Finn et al., 2020). As shown in Panel B of Figure 1, the delayed treatment effect can lead to an accelerated failure phenomenon at the early stage of the survival curve, which violates the proportional hazards assumption for statistical analysis. This phenomenon can diminish treatment efficacy, reduce the power of the study, and make it challenging to interpret the final results (Reck et al., 2016). In other words, studies may ultimately fail if this issue is not adequately and thoroughly taken into account at the study design stage (Ren et al., 2023). Particularly in an adaptive phase II/III seamless design or a group sequential design, delayed treatment effects can lead to wrong go/no-go decisions at the interim analysis. This is attributed to a substantial proportion of events occurring in the accelerated failure stage due to the limitations imposed by the observation period. The following two situations may occur if left unaddressed: first, early termination of an effective treatment due to a low predictive success rate in the final analysis with a fixed sample size; and second, re-estimating an overpowered sample size when adjustments based on interim analysis results are permitted. Similar situations can arise in phase II/III seamless designs. Figure 2 demonstrates the simulated impact of delayed treatment effects on the conditional and predictive power at the interim analysis of group sequential designs. It is evident that increasing delay time results in decreased conditional and predictive power. Another point worth noting in Figure 2 is that solely increasing the number of interim analysis events has limited efficacy in compensating for the loss caused by the delayed treatment effect. Thus, this action is insufficient to address the issue. In light of the aforementioned considerations, the primary objective of this paper is to find targeted methods that improve the decision success rate of the interim analysis in the presence of delayed treatment effects in phase II/III seamless designs or group sequential designs.

In contrast to the existing body of research on delayed treatment effects that predominantly focuses on fixed designs and final analysis, there is a notable dearth of methods specifically tailored for interim analysis. Moreover, the majority of existing methods rely on the weighted log-rank test statistic and its extension methods (Hasegawa, 2016; Yang, 2019; Prior, 2020). Despite disregarding the complex calculation and poor interpretability associated with the weighted log-rank test statistic, there is still a deficiency in its application. Specifically, in phase III confirmatory tumor clinical trials, the log-rank test serves as the primary method for analyzing time-to-event endpoints, regardless of whether the proportional hazard assumption is satisfied, while the weighted log-rank test statistic is often only used as a sensitivity analysis. Consequently, we shift our research direction toward improving the go/no-go decision success rate of the interim analysis. To address this research issue, we propose a comprehensive set of methods and procedures. We first detect the number and locations of change points in the survival curve, estimate the hazard function values for each segment of the survival curve based on these change points, then estimate the treatment effect size, and finally apply it to go/no-go decision indicators such as conditional power, predictive power, or the probability of success.

A change point is defined as a specific juncture at which the probability density function of random variables changes (Hinkley, 1970). The survival curve typically has one or more change points when the proportional hazards assumption is violated. Panel B in Figure 1 illustrates an example where the two survival curves overlap in the first 5 months due to a delayed treatment effect. Notably, a distinct change point can be observed in the red dashed survival curve, as indicated by the circle in the figure. Various statistical methods have been proposed for change point detection and hazard function estimation. Matthews and Farewell (1982) used the maximum likelihood function to find a single change point in the survival curve at an unknown time. Goodman et al. (2011) proposed using the maximum profile likelihood function to detect multiple change points and hazard functions in a piecewise exponential model. They also applied the Wald-type statistic for sequential testing of the statistical significance of these change points. The sequential testing means testing the k^th^ change point only when the kth-1 change point is significant. Moreover, they used a decreasing alpha spending function to control the type I error rate, which is similar to the approach proposed by Lan and DeMets (1983) in group sequential design. Matthews et al. (1985) suggested using the score statistic to test the significance of change points. He et al. (2013) proposed a sequential testing procedure using the likelihood ratio statistic to identify multiple change points. In addition to frequentist methods, Bayesian methods have also been used, with Raftery and Akman (1986) first introducing a Bayesian framework that focuses on the Poisson process with a single change point. Arjas and Gasbarra (1994) used a Gibbs sampler to identify multiple change points. Chapple et al. (2020) applied the Markov Chain Monte Carlo (MCMC) method and a reversible jump algorithm to determine the number and location of change points. Additionally, a non-parametric approach was proposed by Müller and Wang (1994), who used a non-parametric smoothing technique to detect change points. Although the aforementioned methods can be used for change point detection and hazard function estimation, none of them mentioned clinical phase II/III seamless design or group sequential design, and there are few methods related to clinical trial practice. Therefore, we modify some frequentist and Bayesian methods, and provide recommendations on the order of searching for multiple change points to ensure their suitability for our research purposes. Furthermore, we propose two non-parametric methods based on the Kaplan–Meier estimator and the area under the survival curve, respectively. These two non-parametric methods demonstrate comparable performance to the parametric methods under certain simulation scenarios while reducing the complexity of change point detection. Next, while the aforementioned methods allow for testing the statistical significance of the detected change points, most of them are based on the hazard ratio (HR) and are recommended to be used in the unblinded analysis. However, in real clinical trials, interim analysis may need to be conducted without unblinding. Therefore, we propose a sequential testing approach based on the ratio of the log-likelihood function, which differs from the method proposed by He et al. (2013) and can be applied to test change points in both blinded and unblinded analyses. Finally, by calculating the average hazard ratio (AHR), we can apply the change points and hazard functions to the efficacy estimation. The AHR is then incorporated into go/no-go decision indicators to assist us in making decisions. In addition to the traditional AHR (Kalbfleisch and Prentice, 1981), we also propose a new AHR calculation method whose weighting considers both the influence of time and the number of events. Our proposed analysis procedure involves both blinded and unblinded interim analyses. The main distinction is that unblinded analysis allows for an additional test for the proportional hazards assumption prior to commencing the analysis procedure. If the test result is statistically significant, it indicates that the proportional hazards assumption may not hold. We proceed to detect change points and estimate hazard functions. Otherwise, the interim analysis is conducted directly without using the proposed analysis procedure. Since existing methods for testing the proportional hazards assumption are primarily based on the HR (Moore, 2016), estimating it under the blind condition is complex and requires some strong assumptions. As a result, we skip this step and directly detect change points in the blinded analysis. Moreover, since determining an appropriate time for interim analysis when the proportional hazards assumption does not hold is a topic worth discussing, we propose a maximum interim information design. This flexible and simple-to-implement method considers both event-driven and calendar-driven factors when selecting the interim analysis time.

The subsequent sections of this paper are organized as follows: Section 2 introduces the methods for detecting and testing change points, estimating the effect size, and choosing the analysis time for the interim analysis. To depict the proposed analysis procedure more clearly, we also provide an analysis flowchart at the end of this section. In Section 3, Section 4, Section 5, we compare the performance of the proposed methods with the unadjusted traditional method in various scenarios and a real case. The discussion remarks are summarized in the last section.

## 2 Methods

### 2.1 Change point detection methods

#### 2.1.1 Frequentist approach

The survival time and censoring time of N subjects are denoted as $X=\left\{x_{1},x_{2}\ldots x_{N}\right\}$ and $C=\left\{c_{1},c_{2}\ldots c_{N}\right\}$ , respectively. Let $T=\left\{t_{1},t_{2}\ldots t_{N}\right\}$ represent the observed time, where $t_{i}=\min\left(x_{i},c_{i}\right)$ . Assuming that the hazard function $h\left(t\right)$ in the survival curve has a total of K change points, with $\tau_{k}$ denoting the time of the $k^{th}$ change point, where $\tau_{k}\in \left(0,+\infty \right)$ , we adopt the piecewise exponential model to describe the distribution of the random variable T , which is modeled by

$$p\left(t_{i}|\lambda_{k},\tau_{k}\right)=\left\{\begin{array}{l} \varphi_{1}\begin{array}{c} \begin{array}{cc} \lambda_{1}^{\delta_{i}}\exp\left(-\lambda_{1}t_{i}\right) & t_{0}\leq t_{i}<\tau_{1} \end{array} \end{array}\\ \vdots \\ \begin{array}{cc} \varphi_{K+1}\lambda_{K+1}^{\delta_{i}}\exp\left(-\lambda_{K+1}t_{i}\right) & \tau_{K}\leq t_{i}<t_{T} \end{array} \end{array}\right.,$$

where $\delta_{i}=I\left(x_{i}\leq c_{i}\right)$ . $\varphi_{k}$ is a constant, with $\varphi_{1}=$ 1 and $\varphi_{k}=\exp\left[-\lambda_{1}\left(\tau_{1}-\tau_{0}\right)-\lambda_{2}\left(\tau_{2}-\tau_{1}\right)-\ldots -\lambda_{k-1}\left(\tau_{k-1}-\tau_{k-2}\right)+\lambda_{k}\tau_{k-1}\right]$ when k>1 , making $\int_{0}^{\infty }p\left(t_{i}|\lambda_{k},\tau_{k}\right)dt_{i}=1$ . In this model, the hazard function $h\left(t\right)$ for each piece is assumed to be constant and represented by the hazard rate $\lambda_{k}$ . $t_{0}$ and $t_{T}$ are two constants. Typically, $t_{0}$ is set to 0 and $t_{T}$ is set to the maximum planned follow-up period. The log-likelihood function is then defined as follows:

$$lnL\left(\lambda_{1},\ldots ,\lambda_{K+1},\tau_{1},\ldots ,\tau_{K}\right)=$$

$$\sum_{k=1}^{K+1}\left[U\left(\tau_{k}\right)-U\left(\tau_{k-1}\right)\right]\ln\lambda_{k}-\sum_{k=1}^{K+1}\sum_{i=1}^{N}\left[V\left(\tau_{k}\right)-V\left(\tau_{k-1}\right)\right]\lambda_{k},$$

where $U\left(t\right)=\sum_{i=1}^{N}I\left(t_{i}\leq t,\delta_{i}=1\right)$ and $V\left(t\right)=t_{i}\wedge t$ . Estimates of parameters $\lambda_{k}$ and $\tau_{k}$ can be obtained using the maximum profile likelihood function. This process involves first holding $\tau_{k}$ fixed and then maximizing $\lambda_{k}$ to obtain

$$\lambda_{k}=\frac{U\left(\tau_{k}\right)-U\left(\tau_{k-1}\right)}{\sum_{i=1}^{N}\left[V\left(\tau_{k}\right)-\tau_{k-1}\right]I\left(t_{i}>\tau_{k-1}\right)}.$$

The log-likelihood function is changed by substituting the given equation of $\lambda_{k}$ into

$$LnL\left(\tau_{1},\ldots ,\tau_{K}\right)=\sum_{k=1}^{K+1}\left[U\left(\tau_{k}\right)-U\left(\tau_{k-1}\right)\right]\ln\left\{\frac{U\left(\tau_{k}\right)-U\left(\tau_{k-1}\right)}{\sum_{i=1}^{N}\left[V\left(\tau_{k}\right)-\tau_{k-1}\right]I\left(t_{i}>\tau_{k-1}\right)}\right\}.$$

We obtain $\tau_{k}$ by maximizing $LnL\left(\tau_{1},\ldots ,\tau_{K}\right)$ and then substituting it into the equation to obtain $\lambda_{k}$ . When the survival curve has only one change point, there are two approaches to determine $\tau_{k}$ : either by considering a list of several possible combinations of $\tau_{k}$ and choosing the one that maximizes the log-likelihood function or by directly solving for $\tau_{k}$ using numerical optimization techniques. However, in situations where there are multiple change points or the number of change points is unknown, we recommend following the following steps outlined to detect the number of change points. For example, if there is at least one change point in the first 10 months and the change point observed in the 4th month is both the maximum of the log-likelihood function and statistically significant, we proceed to detect the most likely second change point within the intervals of the 1st to the 4th and the 4th to the 10th month. Assume that it is in the 6th month. We then test the statistical significance of this second change point. If the result is significant, we continue to detect the third change point within the intervals of the 1st to the 4th, the 4th to the 6th, and the 6th to the 10th month. On the other hand, if the statistical significance of the second change point is not achieved, we consider the survival curve to have only one change point. An alternative method is to use enumeration, which lists all possible combinations of change points. However, this method may be impractical when there are multiple change points as the number of combinations can become excessively large.

#### 2.1.2 Bayesian approach

In a Bayesian framework, the posterior density is proportional to the product of the likelihood function and the prior density. Specifically,

$$p\left(\lambda_{1},\ldots ,\lambda_{K+1},\tau_{1},\ldots ,\tau_{K},K|D\right)$$

$$\propto L\left(D|\lambda_{1},\ldots ,\lambda_{K+1},\tau_{1},\ldots ,\tau_{K},K\right)\prod_{k=1}^{K+1}p\left(\lambda_{k}|\alpha_{k},\beta_{k},\tau_{k},K\right)\prod_{k=1}^{K}p\left(\tau_{k}|\gamma_{k},K\right)p\left(K|\theta \right),$$

where D represents the phase II data on a seamless design or the interim data on a group sequential design. It is assumed to follow the same piecewise exponential model, as described in the previous section. The prior distribution of parameter $\lambda_{k}$ is assumed to follow $Gamma\left(\alpha_{k},\beta_{k}\right)$ with an inverse scale parameter, and $\lambda_{k}$ is close to a non-informative prior when $\alpha_{k}\leq 0.01$ and $\beta_{k}\leq 0.01$ . The prior distribution of $\tau_{k}$ follows $Uniform\left(\gamma_{k-1},\gamma_{k}\right)$ . The random variable K is assumed to follow a truncated Poisson prior, and $p\left(K\right)=\frac{\theta^{K}}{\sum_{k=1}^{K^{\max}}\frac{\theta^{k}}{k!}}\frac{\left(K^{\max}-K\right)!}{K^{\max}!}$ , where the hyperparameter θ represents the average number of change points in a unit time. Its value can be chosen based on specific circumstances. A larger value of θ indicates a higher frequency of change point occurrences, and *vice versa*. $K^{\max}$ is the maximum predictive number of change points up to $\frac{N}{2}$ . $p\left(K|\theta \right)=1$ when $K^{\max}=1$ . The number of change points is accepted with Metropolis–Hastings probability $\min\;\left(1,M\right)$ , where $M=\frac{p\left(\lambda_{1},\ldots ,\lambda_{K+1},\tau_{1},\ldots ,\tau_{K},K|D\right)}{p\left(\lambda_{1},\ldots ,\lambda_{K^{*}+1},\tau_{1},\ldots ,\tau_{K^{*}},K^{*}|D\right)}\frac{p\left(K,K^{*}\right)}{p\left(K^{*},K\right)}$ and $K^{*}$ is another possible number of change points other than K . Typically, $p\left(K,K^{*}\right)=p\left(K^{*},K\right)$ . The posterior distribution of parameters based on the piecewise exponential model is expressed as

$$p\left(\lambda_{1},\ldots ,\lambda_{K+1},\tau_{1},\ldots ,\tau_{K},K|D\right)\propto$$

$$\prod_{k=1}^{K+1}\lambda_{k}^{U\left(\tau_{k}\right)-U\left(\tau_{k-1}\right)+\beta_{k}}e^{-\lambda_{k}\left[\sum_{i=1}^{N}V\left(\tau_{k}\right)-\sum_{i=1}^{N}V\left(\tau_{k-1}\right)+\alpha_{k}\right]}\;\prod_{k=1}^{K}\frac{1}{\gamma_{k}-\gamma_{k-1}}\frac{\theta^{K}}{\sum_{k=1}^{K^{\max}}\frac{\theta^{k}}{k!}}\frac{\left(K^{\max}-K\right)!}{K^{\max}!}.$$

The model’s parameters can be estimated using the MCMC method.

#### 2.1.3 Non-parametric approach

**FIGURE 3:**
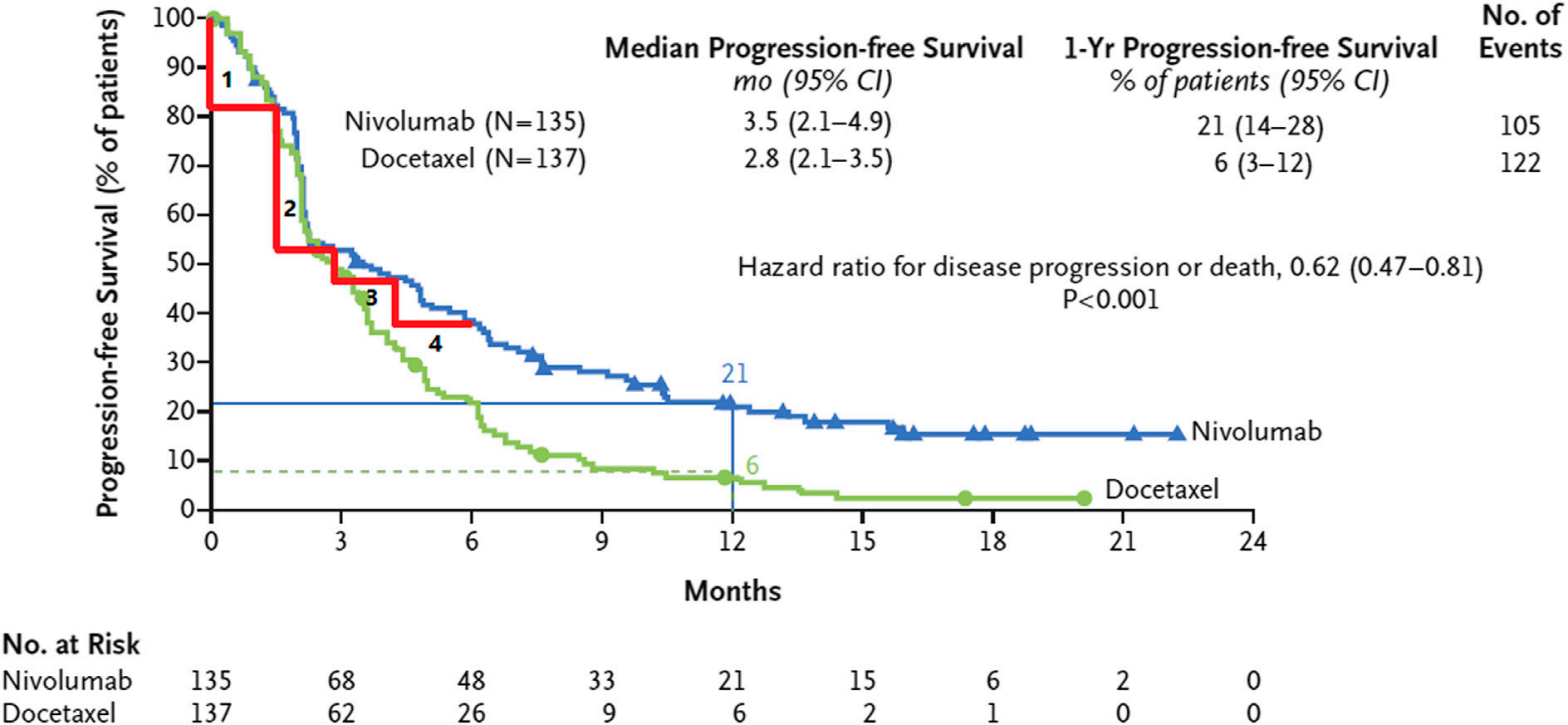
The survival curve of nivolumab has one change point in the 3rd month after the first administration and is divided into four subintervals of equal length.

The frequentist and Bayesian approaches have a disadvantage which is the fact that their calculation processes can become overly complex. To overcome this limitation, we propose two non-parametric methods. The idea of the non-parametric approach is very intuitive, as shown in Figure 3 (Brahmer et al., 2015), which describes that the survival curve of the nivolumab group has one change point near the 3rd month after the first administration. This implies that there are different hazard rates before and after the change point. Our first approach involves dividing the survival curve into multiple time subintervals of equal length. We calculate the area under the survival curve for each time subinterval and identify the subinterval that has the largest change in comparison to the previous subinterval. The starting point of this subinterval is chosen as the change point. Taking the identification of one change point as an example, we first predict a range of the change point, such as from the 1st to the 5th month after the first administration. The survival curve is divided into equal-length time subintervals, for example, from the 1st to the 2nd month, from 1.1th to the 2.1th month, or from the 1.2th to the 2.2th month, denoted by $I_{1-2}$ , $I_{1.1-2.1}$ , and $I_{1.2-2.2}$ , respectively. For each subinterval, we calculate the area under the survival curve and subtract it from the area of its neighboring subinterval, such as $I_{1.1-2.1}$ - $I_{1-2}$ and $I_{1.2-2.2}$ - $I_{1.1-2.1}$ . It should be noted that the area under the survival curve of $I_{t_{1}-t_{2}}$ is equal to $\int_{t_{1}}^{t_{2}}\left(S\left(t\right)-S\left(t_{2}\right)\right)dt$ , which indicates that we only need to calculate part of the area under the survival curve rather than the entire area. The final step is to sort all the results from the previous calculations and identify the two neighboring subintervals with the largest difference in area. Referring to Figure 3 for illustration purposes, we divide the survival curve of the nivolumab group into four subintervals. By comparing the difference in the area under the curve, the largest difference lies between the 2nd and 3rd subintervals, which corresponds to the 3rd month after the first administration as the change point. Another non-parametric method, similar to the area method, uses the Kaplan–Meier estimator as the criterion instead of the area. Additionally, the lengths of the subintervals are not necessarily equal during the division process. In this method, we first list the possible change point range, such as from the 1st to the 5th month. Next, we divide the survival curve into two subintervals, for example, from 0 to the 1st month and from the 1st to the 6th month. We calculate the logarithmic Kaplan–Meier estimator for each subinterval, $\ln\hat{S}{\left(t\right)}_{0-1}=\sum_{t_{i}=0}^{1}\ln\left(1-\frac{d_{i}}{r_{i}}\right)$ and $\ln\hat{S}{\left(t\right)}_{1-6}=\ln\hat{S}{\left(t\right)}_{0-6}-\ln\hat{S}{\left(t\right)}_{0-1}$ . Finally, we divide by the length of each subinterval and subtract, resulting in $\frac{\ln\hat{S}{\left(t\right)}_{1-6}}{5}-\frac{\ln\hat{S}{\left(t\right)}_{0-1}}{1}$ . This process is repeated, and $\frac{\ln\hat{S}{\left(t\right)}_{1.1-6}}{4.9}-\frac{\ln\hat{S}{\left(t\right)}_{0-1.1}}{1.1}$ and $\frac{\ln\hat{S}{\left(t\right)}_{1.2-6}}{4.8}-\frac{\ln\hat{S}{\left(t\right)}_{0-1.2}}{1.2}$ are calculated until $\frac{\ln\hat{S}{\left(t\right)}_{5-6}}{1}-\frac{\ln\hat{S}{\left(t\right)}_{0-5}}{5}$ is reached. The boundary point between two intervals with the largest interval difference is chosen as the change point. After identifying the change points, the hazard rates can be estimated using either frequentist or Bayesian methods. The length of the time subinterval can be adjusted as needed; narrower subintervals typically provide a more accurate change point result. However, through simulations in the subsequent sections, we find that a precision of 0.5 months is usually sufficient, and there is limited practical meaning in having subintervals narrower than 0.5 months.

### 2.2 Hypothesis testing for change points

After identifying the change points, researchers may want to know which of these change points are statistically significant. In addition to the methods introduced in Section 1, we propose a sequential likelihood ratio test that is applicable to both blinded and unblinded analyses. This test consists of the following three steps to assess whether there is one change point or no change point.

I. Use a frequentist, Bayesian, or non-parametric approach to find one change point in real trial data. Calculate the log-likelihood function value based on the piecewise exponential model for this change point, denoted as $L_{1CP}$ . Calculate the log-likelihood function value based on the exponential distribution without any change points in the same data, denoted as $L_{0CP}$ . Derive the log likelihood ratio statistic as $R_{1}$ = $L_{1CP}$ / $L_{0CP}$ .
II. Simulate 1000 samples of data using the hazard function parameters estimated from the exponential distribution without any change points, which were obtained in the previous step. Each sample has the same sample size as the real trial. Repeat step I for each sample, resulting in 1000 likelihood ratio statistics, $R_{2}^{n}=L_{1CP}^{n}/L_{0CP}^{n}$ , where n=1,…,1000 .
III. Substitute $R_{1}$ in $R_{2}^{n}$ and sort them in ascending order. If $R_{1}$ is greater than (1- α ) of the $R_{2}^{n}$ values, such as α = 0.1, it is considered that there is one change point. Otherwise, it is concluded that no change point exists.

By repeating the same sequential test steps, it is possible to determine the presence of two change points beyond one. If two change points are confirmed, we can proceed to test whether there are three or more change points. In general, this sequential test does not require adjusting the type I error rate. However, to avoid the over-fitted issue with a large number of change points, we can refer to the decreasing alpha spending function proposed by Goodman et al. (2011). It is important to note that there may be cases where the hazard function changes significantly after a change point, yet the proportional hazards assumption still holds, such as in Panel F of Figure 1. We simulate and discuss the impact of such a case in Section 3, Section 4.

### 2.3 Treatment effect size estimation

To estimate the treatment effect using the change points and hazard functions obtained from the previous steps, a common approach is to use the AHR proposed by Kalbfleisch and Prentice (1981). For any two treatment groups, the AHR can be calculated as follows:

$$AHR1=\frac{\int_{0}^{\infty }h_{T}\left(t\right)S_{C}^{p}\left(t\right)S_{T}^{p}\left(t\right)dt}{\int_{0}^{\infty }h_{C}\left(t\right)S_{C}^{p}\left(t\right)S_{T}^{p}\left(t\right)dt},$$

where $h_{T}\left(t\right)$ and $h_{C}\left(t\right)$ denote the hazard functions of the test and control groups, respectively. $S_{C}^{p}\left(t\right)S_{T}^{p}\left(t\right)$ is the weighted survival function. When assuming that the survival data follow a piecewise exponential model with K change points, the AHR can be expressed as

$$AHR1=\frac{\int_{0}^{\tau_{1}}h_{T}\left(t\right)S_{C_{1}}^{p}\left(t\right)S_{T_{1}}^{p}\left(t\right)dt+\ldots +\int_{\tau_{K}}^{\infty }h_{T}\left(t\right)S_{C_{K+1}}^{p}\left(t\right)S_{T_{K+1}}^{p}\left(t\right)dt}{\int_{0}^{\tau_{1}}h_{C}\left(t\right)S_{C_{1}}^{p}\left(t\right)S_{T_{1}}^{p}\left(t\right)dt+\ldots +\int_{\tau_{K}}^{\infty }h_{C}\left(t\right)S_{C_{K+1}}^{p}\left(t\right)S_{T_{K+1}}^{p}\left(t\right)dt}.$$

The aforementioned weighting method has the disadvantage of being complex to calculate when there is more than one change point, and it is also challenging to choose the value of p . As an alternative, there is another weighting method that assumes that the HR of the $k^{th}$ subinterval follows a log-normal distribution, with $\ln\left(h_{T_{k}}\left(t\right)/h_{C_{k}}\left(t\right)\right)$ as the mean and $r/E_{k}$ as the standard deviation, where $E_{k}$ represents the number of events in the $k^{th}$ subinterval. r equals 2 when the two groups are in a 1:1 allocation ratio. This method uses the inverse standard deviation square to derive the AHR. It calculates the overall hazard function as the weighted sum of constant hazard rates for each subinterval in the survival curve, like $h\left(t\right)=\sum_{k=1}^{K+1}W_{k}\lambda_{k}$ , where the weighting factor $W_{k}=\frac{{1/\sigma }_{k}^{2}}{\sum_{k=1}^{K+1}{/\sigma }_{k}^{2}}=\frac{E_{k}/4}{\sum_{k=1}^{K+1}E_{k}/4}$ . Consequently, the equation for this alternative AHR is

$$AHR2=\frac{E_{1}\lambda_{T_{1}}+\ldots +E_{K+1}\lambda_{T_{K+1}}}{E_{1}\lambda_{C_{1}}+\ldots +E_{K+1}\lambda_{C_{K+1}}}.$$

Although the weights assigned to AHR2 are easily chosen and assume equal weight for each event, they only consider the number of events and disregard the influence of the duration of each subinterval in the survival curve. For instance, in a survival curve with one change point in the 3rd month after the first administration, there exists an accelerated failure phase before the change point. It is assumed that 100 events are observed both before and after the change point, respectively. Both subintervals are equally weighted when using the AHR2. However, the observation duration for the first 100 events is only 3 months, considerably shorter than the duration after the change point, and there are still large amounts of censored data after the change point that is not included in the weights. Considering these factors, we propose another weighting method that takes into account the impact of both time and the number of events, which is called the time- and event-weighted HR (TEHR). It is calculated as follows:

$$TEHR=\frac{E_{1}\int_{0}^{\tau_{1}}\lambda_{T_{1}}dt+\ldots +E_{k+1}\int_{\tau_{k}}^{T}\lambda_{T_{K+1}}dt}{E_{1}\int_{0}^{\tau_{1}}\lambda_{C_{1}}dt+\ldots +E_{k+1}\int_{\tau_{k}}^{T}\lambda_{C_{K+1}}dt}.$$

One benefit of the TEHR is that the duration of time used in the AHR1 and the number of events used in the AHR2 are taken into account when assigning weights. Both the AHR and TEHR can be calculated directly in unblinded analysis. The challenge is in the estimation of the hazard function for each treatment group without unblinding. One approach is to use the EM algorithm to estimate the hazard function. The other approach is to make certain assumptions. For example, it is assumed that the blind combined hazard rate $\lambda_{k}$ and the predictive hazard ratio $HR_{k}$ in the $k^{th}$ subinterval equal $\frac{\lambda_{T_{k}}+\lambda_{C_{k}}}{2}$ and $\frac{\lambda_{T_{k}}}{\lambda_{C_{k}}}$ , respectively. Then, we can obtain $\lambda_{C_{k}}=\frac{2\lambda_{k}}{HR_{k}+1}$ and $\lambda_{T_{k}}=\frac{2\lambda_{k}HR_{k}}{HR_{k}+1}$ to calculate the AHR or TEHR. Although this method is relatively straightforward to implement, it only allows estimation of $\lambda_{k}$ under the blind condition and we need to predict the value of $HR_{k}$ . When the actual value of $HR_{k}$ significantly deviates from its predictive value, the results of AHR and TEHR may be inaccurate.

### 2.4 Maximum interim information design

When planning an interim analysis in a group sequential design, the interim analysis time is usually determined using either an event- or calendar-driven approach. In an event-driven design, the interim analysis is triggered when a predetermined number of events is observed. A calendar-driven design refers to conducting the interim analysis after a pre-specified time has arrived. Researchers typically choose one of these approaches as the criteria for initiating the interim analysis. For example, a tumor immunotherapy trial plans to conduct an interim analysis when 150 events are observed. In the case where the proportional hazards assumption is established, 150 events are expected to be observed in the 18th month after the first administration. Hence, either event-driven or calendar-driven criteria could be used to determine the interim analysis time. However, if a delayed treatment effect is present, the hazard rate would be higher before the change point and decrease afterward. Consequently, it is possible to observe 150 events before the 18th month. On the other hand, if the hazard rates of both groups are relatively low before the change point but rise rapidly in the control group after the change point, fewer than 150 events may be observed in the 18th month. To this end, we propose a flexible method for determining the interim analysis time that considers event- and calendar-driven information. The approach selects the later of the two criteria as the interim analysis time. In the previously described first scenario, the interim analysis would be conducted in the 18th month after the first administration, where there would be more than 150 events due to the presence of a delayed treatment effect. This approach, which selects the interim analysis time with the larger information fraction between event- and calendar-driven, is referred to as the maximum interim information design.

### 2.5 Analysis flowchart

**FIGURE 4:**
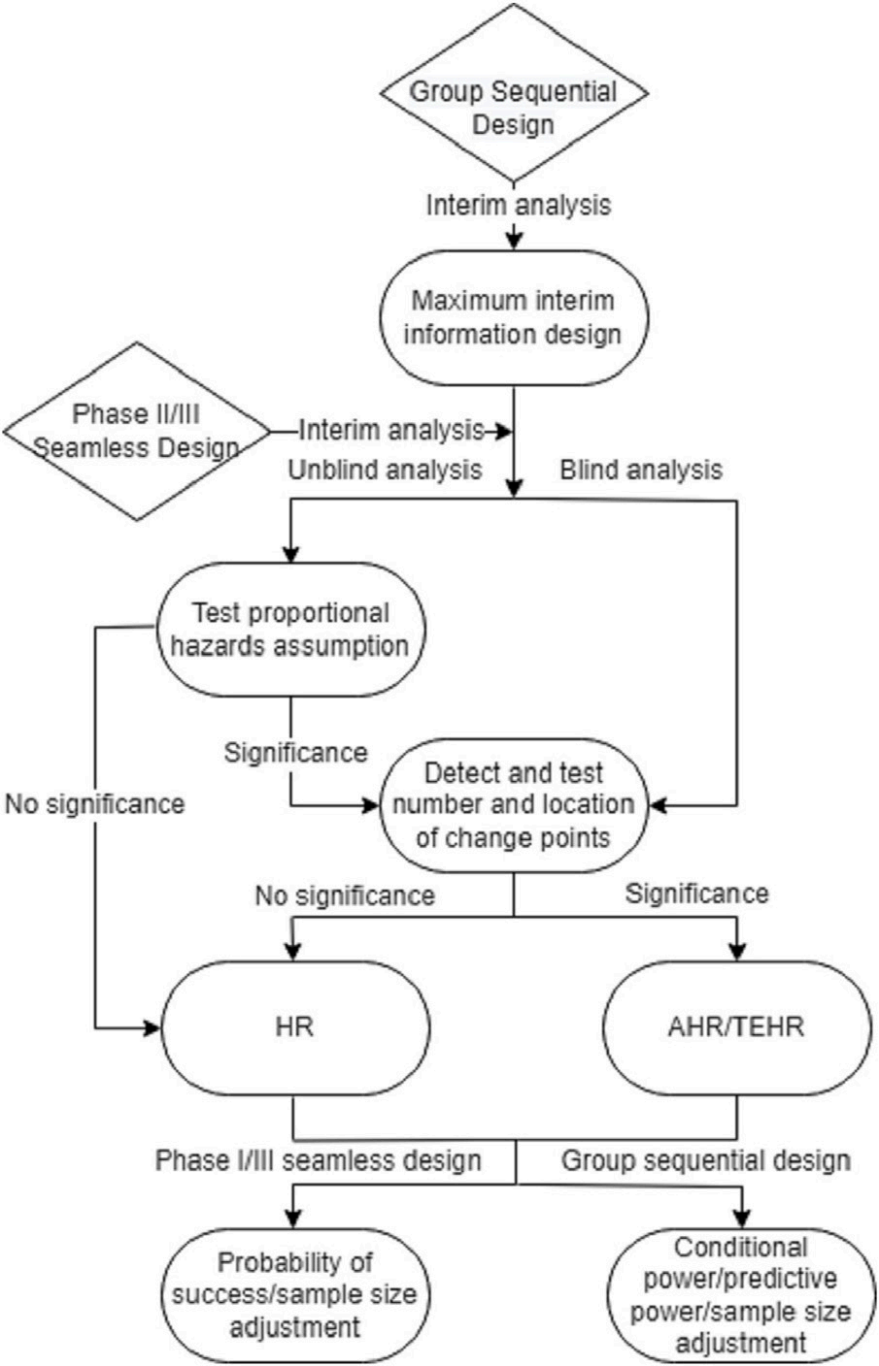
Analysis flowchart for phase II/III seamless design and group sequential design when there may be a delayed treatment effect.

Figure 4 introduces a flowchart that provides an outline of the proposed analysis procedure for the phase II/III seamless design and the group sequential design in the presence of a potential delayed treatment effect. This flowchart can serve as a useful reference when implementing the proposed analysis procedure.

## 3 Simulation

In this section, we first conducted a comparative simulation for different change point detection methods under various sample sizes and data maturities. Our simulations focused on a scenario with a single treatment group and assumed that there would be only one change point, which was in the 2nd, 3.5th, or 5th month after the first administration, respectively. The sample sizes were 200, 300, and 500, while the censoring rates were set at 0%, 20%, or 50%. We denoted the hazard rates of the survival curve before and after the change point as $\lambda_{1}$ and $\lambda_{2}$ . In all scenarios, $\lambda_{1}$ remained constant at 0.1, while $\lambda_{2}$ equals 0.05, 0.067, or 0.1, respectively. The simulated data were sampled from the piecewise exponential model based on $\lambda_{1}$ and $\lambda_{2}$ . Additionally, we evaluated the relationship between the sample size and the change point estimation accuracy by simulating results with event numbers of 100, 200, 300, 500, 1000, and 2000 without any censored data. The comparison included the frequentist method, the Bayesian method, and two non-parametric methods introduced in the previous subsection. The range of change point selection for the frequentist method and two non-parametric methods was from the 1st to the 8th month, with an interval of 0.5 months. This meant that there were 15 possible choices for the change point. The hazard rates for the non-parametric methods were estimated using the equation for $\lambda_{k}$ in Section 2.1.1. In the Bayesian method, the prior distribution for parameters $\lambda_{1}$ and $\lambda_{2}$ followed $Gamma\left(0.01,0.01\right)$ with an inverse scale parameter and τ followed $Uniform\left(1,8\right)$ . Furthermore, we simulated a clinical trial with two treatment groups to evaluate the performance of the proposed sequential likelihood ratio test. The test objects were the test group, the control group, and the pooled data on the two groups. We used Cox regression with an interaction term of time and the treatment group as a benchmark. The simulation encompassed a total of 300 subjects, with 150 subjects in each treatment group. It was assumed that the test group had one change point, with the location and hazard rates before and after the change point being the same as the settings in the previous simulations. The control group had no change point, and its hazard rate was fixed at 0.1.

Next, to assess the impact of the proposed analysis procedure on the decision success rate at the unblinded interim analysis, we conducted a simulation of a double-blind, randomization, placebo-controlled phase III clinical trial. The trial had two treatment groups. Overall survival (OS) was the primary efficacy endpoint. The estimated median OS for the test group was 12 months ( $\lambda_{1}$ = 0.057762), while the control group had a median OS of 8 months ( $\lambda_{2}$ = 0.086643). The significance level was set at 0.05. The recruitment duration was 12 months, and the follow-up duration was 24 months. A total of 260 events were required to achieve 90% statistical power, and each treatment group was planned to enroll 150 subjects in a 1:1 ratio. An unblinded interim analysis was scheduled to take place after observing 2/3 of the total number of events, which were 174 events. All simulated data were generated from the piecewise exponential model based on the five scenarios in Table 1. There was no change point in Scenario 1, and its true hazard rate was the same as the estimated hazard rate. Scenarios 2, 3, and 5 included one change point occurring between the 2nd and 5th months after the first administration. In scenarios 2 and 3, the hazard rates of the two treatment groups were identical before the change point but differed afterward. Hence, the proportional hazards assumptions were untenable for both scenarios. Scenario 5, discussed in the previous section, represented a special case where both the test and control groups had one change point, but their HRs before and after the change point remained the same. Consequently, the proportional hazards assumption held even though there was one change point. In Scenario 4, we simulated a completely ineffective situation for the test group. We used the same methods as those in the previous simulations to detect change points and estimate hazard rates. The unadjusted interim analysis was used as a comparative reference. Conditional and predictive power were the indicators used to determine the go/no-go decision.

**TABLE 1:** Summary of various scenarios used in the simulations.

Scenario	Actual hazard rate before the change point (median OS)		Actual hazard rate after the change point (median OS)	
	Test group	Active control group	Test group	Active control group
1	0.057762 (12)	0.086643 (8)	0.057762 (12)	0.086643 (8)
2	0.086643 (8)	0.086643 (8)	0.043322 (16)	0.086643 (8)
3	0.086643 (8)	0.086643 (8)	0.057762 (12)	0.086643 (8)
4	0.086643 (8)	0.086643 (8)	0.086643 (8)	0.086643 (8)
5	0.057762 (12)	0.086643 (8)	0.043322 (16)	0.064962 (10.67)

To calculate these two indicators, the clinical success criterion was set to 0.76. The predictive power solely relied on the interim analysis data without incorporating any prior knowledge. The decision threshold was set at 90%, indicating that if the conditional or predictive power at the interim analysis exceeded 90%, it was deemed likely that the trial would succeed in the final analysis without requiring any additional actions, such as sample size re-estimation. To measure whether the decision success rate at the interim analysis was improved, we compared the go/no-go decisions with the final log-rank test results. The treatment effect size at the interim analysis was estimated by the AHR1 and TEHR, with p in the AHR1 set to 0.5. We used the maximum interim information design to decide the interim analysis time in all simulations except for the unadjusted method. In the maximum interim information design, the calendar-driven time was the 20th month after the first administration. Therefore, we chose the later of the occurrence time of the 174th event and the 20th month as the interim analysis time. Additionally, we summarized the average number of events and the interim analysis time when using the maximum interim information design under various scenarios. Each scenario was repeated 1000 times using SAS 9.4 software.

## 4 Results

**FIGURE 5:**
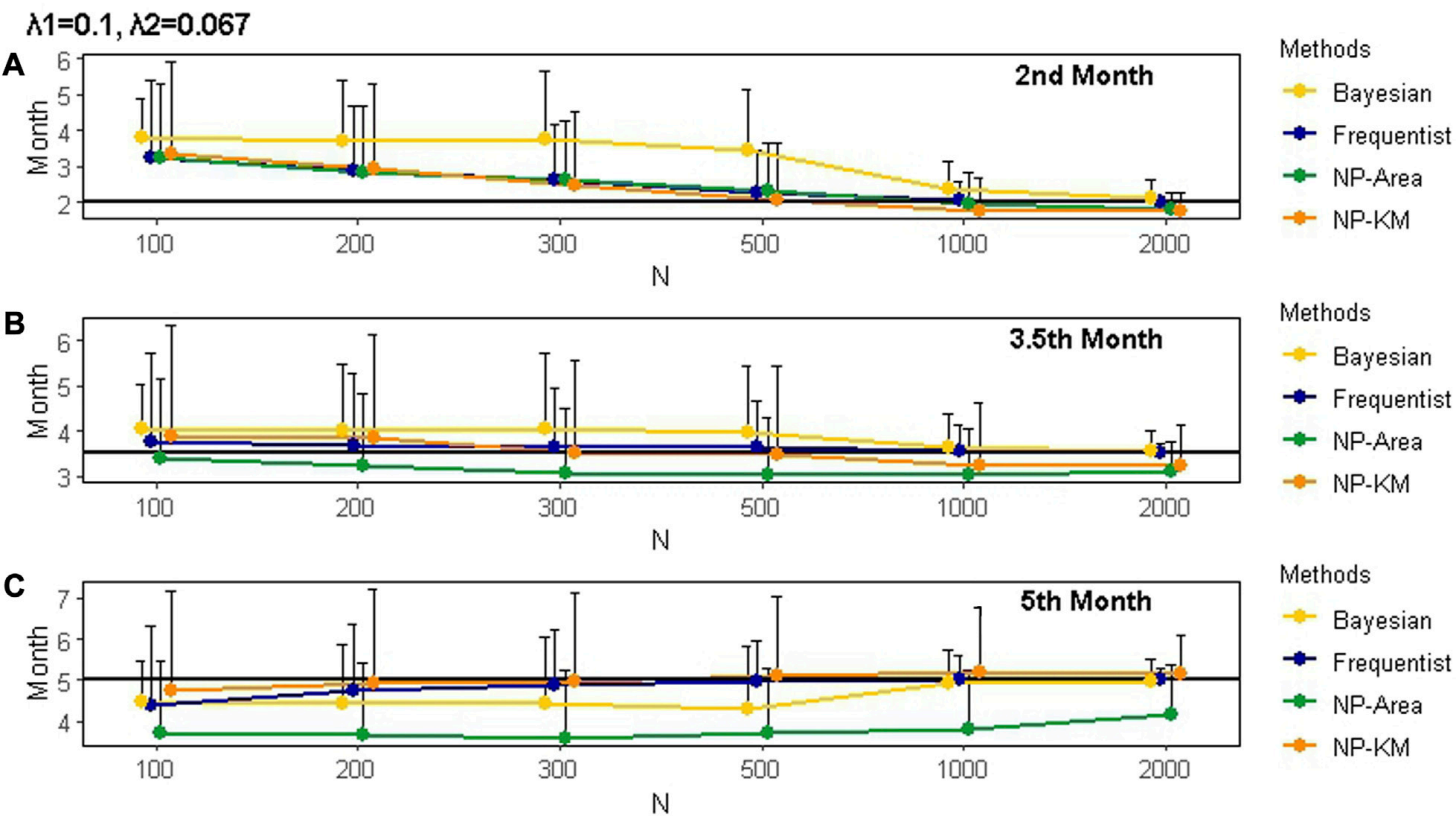
Mean and SD of the actual change points and the change points estimated by different methods under various scenarios for 1000 simulations. Panels **(A)**, **(B)**, and **(C)** represent change points occurring in the 2nd, 3.5th, and 5th months after the first administration, respectively. The black line represents the true value of the change point. The censoring rate is zero. SD is represented by the length of the whisker. The true hazard rates before and after the change points are 0.1 and 0.067, respectively. NP is non-parametric. KM is Kaplan–Meier.

The estimates of change points and hazard rates obtained by different methods, considering varying sample sizes and censoring rates, are summarized in Supplementary Table S1. It is evident that the accuracy of estimates was influenced by the magnitude of the hazard rate gap before and after the change point. Higher accuracy was achieved when the hazard rate gap was larger. In addition, larger sample sizes led to more accurate estimates, and censoring rates also had an impact. The hazard rates estimated by various methods were close to the true value when the estimated change points did not significantly deviate from the true change points. Taking a sample size of 200 and the true change point occurring in the 2nd month after the first administration as an example, we observed that when the estimated change point differed by 0.5 months from the true change point, the estimated hazard rates showed a minor deviation from the true values. Furthermore, when the difference between the estimated and true change points increased to 1 month, as demonstrated in the frequentist method with true hazard rates $\lambda_{1}$ = 0.1 and $\lambda_{2}$ = 0.067 and a censoring rate of 50%, the estimated change point was 3.05. In this case, the impact on the estimated hazard rates remained limited. However, when the difference further increased to 1.5 month, as observed in the Bayesian estimation with true hazard rates $\lambda_{1}$ = 0.1 and $\lambda_{2}$ = 0.05, the bias in the estimated hazard rates became pronounced. This implies that these methods allow for a certain degree of deviation in the change point estimation when estimating hazard rates. This indirectly supports our practice of selecting change points at 0.5-month intervals when using frequentist and non-parametric methods as it demonstrates that a deviation of 0.5 month in the estimate of change points has a limited impact on the estimation of hazard rates before and after the change point. This is also the reason why there were fewer significant differences in the performances of various change point detection methods in subsequent simulations of the decision success rate. Figure 5 further shows the performance of different methods in detecting change points across a wider range of sample sizes. As the sample size increased, the estimated values from all methods gradually approached the true values. The frequentist method performed well in most cases, and when the sample size exceeded 500, its estimated results were nearly identical to the true values, and its SDs were also smaller than those of other methods. Estimates based on the Bayesian method were less accurate than those based on the frequentist method. This can be attributed to two reasons. First, the Bayesian method assumed a uniform distribution for the change point with a wide prior value range, resulting in unsatisfactory change point estimation. Second, the prior distribution of Bayesian parameters used non-informative priors. Its performance can be improved by using an informative prior or reducing the prior value range of the change point. The two non-parametric methods, particularly the one based on the KM estimator, exhibited comparable performance to the frequentist method when the sample size was less than 300 and even outperformed it in certain cases. This is a useful conclusion because, even in phase III trials, the sample size per group for most of them is typically not more than 300. The area-based non-parametric method performed worse than the KM estimator-based non-parametric method, particularly when the change point occurred in the 5th month after the first administration. We observed similar conclusions when $\lambda_{2}$ after the change point was changed to 0.5. Refer to Supplementary Figure S1 for more details. According to the results presented in Supplementary Table S2, the proposed sequential likelihood ratio test outperformed the Cox regression. When we applied the method to test the control or test groups in the absence of the change point, the false positive rate did not exceed 5% in most cases. When there was one change point in the test group, such as $\lambda_{T1}$ = 0.1 and $\lambda_{T2}$ = 0.067, a substantial proportion of results yielded significant findings using the proposed method. Even though the proposed testing method exhibited slightly lower performance as the censoring rate increased, it still outperformed the results of the Cox regression.

For the results of the decision success rate at the interim analysis, α and β in Table 2 refer to the comparison between the conditional or predictive power of the interim analysis and the final analysis results. If the final log-rank test result was statistically significant but the conditional or predictive power was below 90%, 1 was added when calculating β , which was similar to the type II error and indicated a potential incorrect no-go decision. If the final result was not significant but the conditional or predictive power was above 90%, 1 was added in α , which was like the type I error. The summation of incorrect go decisions and incorrect no-go decisions, divided by 1000, respectively, yields the final values of α and β . Typically, the conditional power exceeds the predictive power when it is greater than 0.5, as observed in Scenario 2. On the other hand, when the conditional power is less than 0.5, such as in Scenario 4, it is lower than the predictive power. Hence, in Table 2, we only reported the results of the conditional power and provided simulation results for change points occurring in the 2nd or 5th month after the first administration as the performance of other change points fell between them. See Supplementary Tables S3, S4 for full results.

**TABLE 2:** α and β -values of different methods under various scenarios when the change point is in the 2nd and 5th months.

Change point	Scenario	Unadjusted	MIID only	Frequentist		Bayesian		NP-area		NP-KM	
				AHR1	TEHR	AHR1	TEHR	AHR1	TEHR	AHR1	TEHR
2nd month	1	α = 0.005	α = 0.004	α = 0.011	α = 0.008	α = 0.007	α = 0.008	α = 0.011	α = 0.013	α = 0.014	α = 0.011
		β = 0.31	β = 0.29	β = 0.31	β = 0.33	β = 0.28	β = 0.29	β = 0.25	β = 0.24	β = 0.25	β = 0.22
	2	α = 0	α = 0	α = 0.002	α = 0.004	α = 0.002	α = 0.002	α = 0.002	α = 0.004	α = 0.002	α = 0.003
		β = 0.17	β = 0.15	β = 0.06	β = 0.03	β = 0.07	β = 0.05	β = 0.06	β = 0.04	β = 0.07	β = 0.04
	3	α = 0.004	α = 0.004	α = 0.023	α = 0.036	α = 0.016	α = 0.030	α = 0.018	α = 0.033	α = 0.020	α = 0.027
		β = 0.44	β = 0.40	β = 0.29	β = 0.25	β = 0.32	β = 0.28	β = 0.30	β = 0.25	β = 0.30	β = 0.25
	4	α = 0.003	α = 0.001	α = 0.008	α = 0.011	α = 0.004	α = 0.013	α = 0.006	α = 0.013	α = 0.009	α = 0.011
		β = 0.06	β = 0.06	β = 0.06	β = 0.06	β = 0.06	β = 0.07	β = 0.06	β = 0.06	β = 0.06	β = 0.06
	5	α = 0.006	α = 0.013	α = 0.011	α = 0.015	α = 0.009	α = 0.011	α = 0.007	α = 0.010	α = 0.008	α = 0.012
		β = 0.27	β = 0.16	β = 0.27	β = 0.25	β = 0.29	β = 0.30	β = 0.24	β = 0.22	β = 0.23	β = 0.22
5th month	1	α = 0.005	α = 0.004	α = 0.011	α = 0.006	α = 0.006	α = 0.002	α = 0.007	α = 0.010	α = 0.012	α = 0.006
		β = 0.29	β = 0.27	β = 0.28	β = 0.30	β = 0.29	β = 0.29	β = 0.25	β = 0.23	β = 0.24	β = 0.21
	2	α = 0	α = 0	α = 0.020	α = 0.005	α = 0.017	α = 0.004	α = 0.006	α = 0.008	α = 0.019	α = 0.006
		β = 0.58	β = 0.48	β = 0.17	β = 0.22	β = 0.20	β = 0.24	β = 0.24	β = 0.21	β = 0.20	β = 0.24
	3	α = 0.002	α = 0.001	α = 0.042	α = 0.036	α = 0.031	α = 0.030	α = 0.017	α = 0.037	α = 0.042	α = 0.034
		β = 0.43	β = 0.40	β = 0.27	β = 0.27	β = 0.29	β = 0.28	β = 0.30	β = 0.27	β = 0.28	β = 0.29
	4	α = 0	α = 0	α = 0.007	α = 0.012	α = 0.005	α = 0.008	α = 0.004	α = 0.012	α = 0.008	α = 0.013
		β = 0.05	β = 0.05	β = 0.05	β = 0.06	β = 0.05	β = 0.05	β = 0.05	β = 0.06	β = 0.05	β = 0.06
	5	α = 0.001	α = 0.005	α = 0.015	α = 0.008	α = 0.006	α = 0.004	α = 0.010	α = 0.009	α = 0.013	α = 0.011
		β = 0.24	β = 0.16	β = 0.23	β = 0.23	β = 0.28	β = 0.27	β = 0.23	β = 0.22	β = 0.22	β = 0.21

Note: MIID denotes maximum interim information design. α and β are based on the conditional power. AHR1 is the average HR proposed by Kalbfleisch and Prentice. TEHR is time- and event-weighted HR. NP is non-parametric. KM is Kaplan–Meier.

We first focus on the results in Scenarios 2 and 3 as they had a delayed treatment effect, which is the main issue of this research. In Scenario 3, when we analyzed the data directly without considering the impact of the delayed treatment effect, β exceeded 40% for both 2-month and 5-month delays, which was relatively high. After adjusting the interim analysis time based on the maximum interim information design, the decrease in β was very limited. This finding was consistent with Figure 2, in which we concluded that increasing the number of interim analysis events had little effect on the improvement of power. When using the proposed analysis procedure, regardless of whether it was based on the AHR1 or TEHR, the β -values of most methods were controlled between 0.25 and 0.3, which significantly improved the accuracy of the interim analysis decision. In Scenario 2, the disparity in hazard rates before and after the change point became more pronounced. The unadjusted method exhibited an increase in β from 0.17 to 0.58 as the change point shifted from the 2nd month to the 5th month. Correspondingly, although the various change point detection methods also experienced an increase in β with the delay of treatment efficacy, most of them did not exceed 0.25 when the change point was in the 5th month. This value was less than half of the β -value observed with the unadjusted method. Scenarios 1 and 5 demonstrated that when the proportional hazards assumption held, the maximum interim information design performed best in certain cases. However, in general, there was no significant difference among the methods, with most of them resulting in β between 0.2 and 0.3. In Scenario 4, the β -values were almost the same across all methods.

In all scenarios, the α -values of all methods were controlled below 0.05. The change point detection methods had a slightly larger α than that of the unadjusted method and the maximum interim information design in most scenarios, but the difference was typically no more than 0.03. For example, in Scenario 1, the α -value of the AHR1 of the frequentist method was 0.006 larger than that of the unadjusted method, which indicated that 6 out of 1000 simulated samples had false positive results. This adheres to the rule that α increases as β decreases and *vice versa*. The proposed analysis procedure obtained a significant decrease in β at the cost of a slight increase in α . Similarly, the comparison of the four change point detection methods revealed that, when the sample size was less than 300, the non-parametric methods performed no worse than the frequentist method, and in some scenarios, they even outperformed it, which aligned with previous simulations. However, overall, there was no significant difference in performance observed among the various methods, and the reason has also been explained in the previous simulations.

In the comparison between AHR1 and TEHR, the α -value of TEHR was usually larger than that of AHR1 when the proportional hazards assumption was violated and the change point occurred in the 2nd month. However, when the delayed treatment effect persisted until the 5th month, the α -value of TEHR decreased in comparison to that of the AHR1. The opposite conclusion was observed in β . Therefore, the TEHR is recommended if controlling α is more important, particularly for long-lasting delayed treatment effects. Under the same conditions, if β is more crucial, AHR1 should be chosen.

The number of events adjusted by the maximum interim information design at the interim analysis for various scenarios is shown in Supplementary Table S5. The unadjusted method maintained a fixed number of events at 174. In previous simulations, all change point detection methods selected the interim analysis time based on the maximum interim information design. Therefore, they had the same number of events as the maximum interim information design. In cases where the proportional hazards assumption was held, such as scenarios 1, 4, and 5, different change points had no impact on the number of events. The number of events in scenarios 2 and 3 increased as the delayed treatment effect persisted. This maintained the power of the study to some extent and demonstrated the benefits of the maximum interim information design. Supplementary Table S6 provides a summary of the actual average time of the interim analysis under various scenarios. When there was a delayed treatment effect due to the longer accelerated failure period in the survival curve, the interim analysis time shifted earlier as the number of delayed months increased. In Scenario 5, although the proportional hazards assumption was established, the interim analysis time was postponed. However, as observed in Table 2, this phenomenon had a limited impact on the decision results in Scenario 5. It further reaffirmed that the violation of the proportional hazards assumption was the key factor influencing the accuracy of the analysis and decision-making. In scenarios 1 and 4, where no delayed treatment effect was present, the interim analysis time was not impacted.

## 5 Example

The Bladder1 dataset in the R package survival, which contains information on bladder cancer recurrences, is frequently used by researchers to assess statistical methods. It consists of three treatment groups, namely, thiotepa, pyridoxine, and placebo, comprising 81, 85, and 128 subjects, respectively. The primary endpoint event is defined as the occurrence of bladder cancer recurrence or death due to any reason. Upon plotting the survival curves for these groups, the figure reveals no discernible disparity in the curves during the first 7 or 8 months. However, the decline in the survival curve for the thiotepa group subsequently decelerates, indicating a potential delayed treatment effect. We opted to apply the proposed analysis procedure only to the thiotepa and pyridoxine groups as the placebo group’s survival curve fell between them. We used the log-rank test to compare the thiotepa and pyridoxine groups, yielding a *p*-value of 0.258, suggesting a lack of statistical significance. To convert the trial’s data into interim analysis data, we sorted the data by start time and set the last 30% as censoring, regardless of whether the events were observed. The censoring rate increased to 50.6% as a result. Our intent is to make an accurate go/no-go decision by applying the proposed analysis procedure to the unblinded interim data. To accomplish this, we used frequentist, Bayesian, and two non-parametric methods to detect the change point in the combined data from the 5th to the 9th month after the first administration, maintaining the same settings as the previous simulations for other parameters. Subsequently, we calculated the TEHR, conditional power, and predictive power based on the estimates of the hazard rates before and after the change point for the two groups. The outcomes of the proposed analysis procedure were then compared with those of the unadjusted method. The clinical success criterion was set to 0.8 when calculating the conditional and predictive power. The final results are presented in Table 3.

**TABLE 3:** Interim analysis results were estimated using different methods for the Bladder1 dataset.

Unadjusted	Frequentist	Bayesian	NP-area	NP-KM
HR = 0.761	$\hat{CP}$ = 7	$\hat{CP}$ = 6.2	$\hat{CP}$ = 6	$\hat{CP}$ = 7
CP = 59.33%	TEHR = 0.832	TEHR = 0.785	TEHR = 0.784	TEHR = 0.832
PP = 57.74%	CP = 26.39%	CP = 47.37%	CP = 48.13%	CP = 26.39%
	PP = 30.09%	PP = 47.83%	PP = 48.46%	PP = 30.09%

Note: $\hat{CP}$ denotes the estimate of the change point. TEHR is time- and event-weighted HR. CP and PP denote conditional power and predictive power, respectively. NP is non-parametric. KM is Kaplan–Meier.

The change point detection results obtained through the frequentist and non-parametric methods based on the KM estimator demonstrated higher accuracy than the other two methods. If 50% of this was used as the go/no-go decision criterion, it was evident from the conditional and predictive power that the unadjusted method would have incorrectly decided to continue the trial. In contrast, all values of calculated conditional and predictive power from the proposed analysis process were below 50%, especially the results of the frequentist and non-parametric methods based on the KM estimator, which were exceptionally low.

## 6 Discussion

In the context of phase II/III seamless design and group sequential design, the violation of the proportional hazards assumption can result in two potential outcomes at the interim analysis: overpower and underpower. Overpower occurs when the power of the interim analysis exceeds expectations, while underpower occurs when the power is insufficient due to a delayed treatment effect. Given the impact of the latter on statistical analysis, this paper proposes an analysis procedure that includes detecting the number and location of change points in the survival curve and then using them to estimate the treatment effect size, ultimately improving the decision success rate of the interim analysis. The main benefit of the proposed analysis procedure over the unadjusted method is a significant reduction in the type II error rate at the expense of a slight increase in the type I error rate. The accuracy of the four proposed change point detection methods is influenced by the sample size and censoring rate. In simulations, we found that the frequentist method demonstrated relatively stable performance, while the non-parametric methods, particularly those based on the Kaplan–Meier estimator, outperformed the frequentist method in some scenarios with a sample size of less than 300. All four methods yielded superior results compared to the unadjusted method for decision-making. We introduce TEHR, which incorporates both time and events to estimate efficacy in the presence of delayed treatment effects. In comparison to the commonly used AHR, TEHR has its own advantages, particularly when dealing with multiple change points. We also provide a method for selecting the interim analysis time in the group sequential design, which can limitedly improve the decision success rate. In this section, we discuss important considerations while applying the analysis procedure.

In the previous sections, we summarized the performance of various change point detection methods. However, these conclusions were based on the assumption that the simulated data followed the piecewise exponential model. The selection of the detection method should be guided by the specific characteristics of the data, such as the distribution, sample size, and available prior information. Simulation can assist in identifying an appropriate method for a given scenario. In general, if there is a good understanding of the distribution and an adequate sample size, frequentist methods can be considered. This approach is particularly convenient when the survival curve has only one change point, which is the most common situation in tumor immunotherapy studies. On the other hand, if the distribution is uncertain or the sample size is small, non-parametric methods may be more appropriate. In cases where reliable prior information is available, Bayesian methods can be utilized.

In comparison to AHR2, which applies the same weights for all time intervals, TEHR can be regarded as a weighted approach that incorporates the element of time. In scenarios with a significant treatment delay, TEHR tends to provide a relatively conservative estimate of treatment efficacy between the two groups. Conversely, when there is a shorter delay, TEHR yields a relatively larger estimate of treatment efficacy. This characteristic of TEHR was adequately illustrated through the simulations and the provided example. It is important to note that TEHR is just one type of weighted method that can be used based on the aforementioned rules; it may not be applicable in all situations. Researchers should carefully consider the specific context and characteristics of the study when using TEHR. We did not specifically test for violations of the proportional hazards assumption or the significance of change points while calculating AHR1 or TEHR in the simulations. The results showed that the performance of the proposed analysis procedure was not inferior to that of the unadjusted method when the proportional hazards assumption held, and it exhibited clear advantages when this assumption was violated. This suggests that the proposed analysis procedure can be directly applied to scenarios with no more than one change point without the need to test its statistical significance. Since most real tumor immunotherapy scenarios only involve a single change point, this application condition is particularly useful. It demonstrates the robustness of the proposed analysis procedure while simultaneously reducing the complexity of statistical analysis.

One more issue that has not been discussed is the adjustment and allocation of the type I error rate when using the maximum interim information design. Some traditional methods can still be used to address this issue. For example, the alpha spending function (DeMets and Lan, 1994) can be used to adjust the type I error rate according to the updated interim analysis time. Moreover, the proposed analysis procedure makes decisions based on indicators such as conditional power, predictive power, or probability of success. These indicators can play a decisive role in the phase II/III seamless design. For the group sequential design, in addition to its application in go/no-go decisions, the analysis procedure can also be used in conjunction with the *p*-value obtained at the interim analyses. For instance, if the *p*-value of the interim analysis results cannot reject or accept $H_{0}$ , the proposed analysis procedure can provide complementary insights and a supporting prediction for the final results. The last point to be illustrated is that the analysis procedure is not limited to go/no-go decisions. It can also be applicable to other important objectives, such as sample size re-estimation, and can be extended to various fields that involve delayed treatment effects and change points beyond the scope of tumor immunotherapy.

## Data Availability

Publicly available datasets were analyzed in this study. The dataset Bladder1 of the Survival package used in this study can be found in the CRAN website: https://cran.r-project.org/.

## References

[B1] Arjas E. Gasbarra D. (1994). Nonparametric Bayesian inference from right censored survival data, using the Gibbs sampler. Stat. Sin. 4, 505–524. 10.1007/BF01199902

[B2] Brahmer J. Reckamp K. L. Baas P. Crinò L. Eberhardt W. E. E. Poddubskaya E. (2015). Nivolumab versus docetaxel in advanced squamous-cell non-small-cell lung cancer. N. Engl. J. Med. 373, 123–135. 10.1056/NEJMoa1504627 26028407 PMC4681400

[B3] Chapple A. G. Peak T. Hemal A. (2020). A novel Bayesian continuous piecewise linear log‐hazard model, with estimation and inference via reversible jump Markov chain Monte Carlo. Statistics Med. 39, 1766–1780. 10.1002/sim.8511 32086957

[B4] Chen T.-T. (2013). Statistical issues and challenges in immuno-oncology. J. Immunother. Cancer 1, 18. 10.1186/2051-1426-1-18 24829754 PMC4019889

[B5] DeMets D. L. Lan K. K. G. (1994). Interim analysis: the alpha spending function approach. Stat. Med. 13, 1341–1352. 10.1002/sim.4780131308 7973215

[B6] Farkona S. Diamandis E. P. Blasutig I. M. (2016). Cancer immunotherapy: the beginning of the end of cancer? BMC Med. 14, 73. 10.1186/s12916-016-0623-5 27151159 PMC4858828

[B7] Finn R. S. Ryoo B.-Y. Merle P. Kudo M. Bouattour M. Lim H. Y. (2020). Pembrolizumab as second-line therapy in patients with advanced hepatocellular carcinoma in KEYNOTE-240: a randomized, double-blind, phase III trial. J. Clin. Oncol. 38, 193–202. 10.1200/JCO.19.01307 31790344

[B8] Freeman G. J. Long A. J. Iwai Y. Bourque K. Chernova T. Nishimura H. (2000). Engagement of the PD-1 immunoinhibitory receptor by a novel B7 family member leads to negative regulation of lymphocyte activation. J. Exp. Med. 192, 1027–1034. 10.1084/jem.192.7.1027 11015443 PMC2193311

[B9] Goodman M. S. Li Y. Tiwari R. C. (2011). Detecting multiple change points in piecewise constant hazard functions. J. Appl. Statistics 38, 2523–2532. 10.1080/02664763.2011.559209 PMC3374653 22707842

[B10] Hasegawa T. (2016). Group sequential monitoring based on the weighted log-rank test statistic with the fleming-harrington class of weights in cancer vaccine studies: group sequential monitoring based on the weighted log-rank test statistic with the fleming-harrington class of weights in cancer vaccine studies. Pharm. Stat. 15, 412–419. 10.1002/pst.1760 27353855

[B11] He P. Fang L. Su Z. (2013). A sequential testing approach to detecting multiple change points in the proportional hazards model. Stat. Med. 32, 1239–1245. 10.1002/sim.5605 22933339

[B12] Hinkley D. V. (1970). Inference about the change-point in a sequence of random variables. Biometrika 57, 1–17. 10.1093/biomet/57.1.1

[B13] Hodi F. S. O’Day S. J. McDermott D. F. Weber R. W. Sosman J. A. Haanen J. B. (2010). Improved survival with ipilimumab in patients with metastatic melanoma. N. Engl. J. Med. 363, 711–723. 10.1056/NEJMoa1003466 20525992 PMC3549297

[B14] Kalbfleisch J. D. Prentice R. L. (1981). Estimation of the average hazard ratio. Biometrika 68, 105–112. 10.1093/biomet/68.1.105

[B15] Kudo M. (2019). Pembrolizumab for the treatment of hepatocellular carcinoma. Liver Cancer 8, 143–154. 10.1159/000500143 31192152 PMC6547263

[B16] Lan K. K. G. DeMets D. L. (1983). Discrete sequential boundaries for clinical trials. Biometrika 70, 659–663. 10.2307/2336502

[B17] Larkin J. Hodi F. S. Wolchok J. D. (2015). Combined Nivolumab and ipilimumab or monotherapy in untreated melanoma. N. Engl. J. Med. 373, 1270–1271. 10.1056/NEJMc1509660 26398076

[B18] Matthews D. E. Farewell V. T. (1982). On testing for a constant hazard against a change-point alternative. Biometrics 38, 463–468. 10.2307/2530460 7052152

[B19] Matthews D. E. Farewell V. T. Pyke R. (1985). Asymptotic score-statistic processes and tests for constant hazard against a change-point alternative. Ann. Stat. 13. 10.1214/aos/1176349540

[B20] Melero I. Berman D. M. Aznar M. A. Korman A. J. Pérez Gracia J. L. Haanen J. (2015). Evolving synergistic combinations of targeted immunotherapies to combat cancer. Nat. Rev. Cancer 15, 457–472. 10.1038/nrc3973 26205340

[B21] Mick R. Chen T.-T. (2015). Statistical challenges in the design of late-stage cancer immunotherapy studies. Cancer Immunol. Res. 3, 1292–1298. 10.1158/2326-6066.CIR-15-0260 26644449

[B22] Moore D. F. (2016). Applied survival analysis using R. Cham: Springer International Publishing. 10.1007/978-3-319-31245-3

[B23] Müller H. G. Wang J.-L. (1994). “Change-point models for hazard functions,” in Institute of mathematical statistics lecture notes - monograph series (Hayward, CA: Institute of Mathematical Statistics), 224–241. 10.1214/lnms/1215463127

[B24] Prior T. J. (2020). Group sequential monitoring based on the maximum of weighted log-rank statistics with the Fleming–Harrington class of weights in oncology clinical trials. Stat. Methods Med. Res. 29, 3525–3532. 10.1177/0962280220931560 32522077

[B25] Raftery A. E. Akman V. E. (1986). Bayesian analysis of a Poisson process with a change-point. Biometrika 73, 85–89. 10.1093/biomet/73.1.85

[B26] Reck M. Rodríguez-Abreu D. Robinson A. G. Hui R. Csőszi T. Fülöp A. (2016). Pembrolizumab versus chemotherapy for PD-L1–positive non–small-cell lung cancer. N. Engl. J. Med. 375, 1823–1833. 10.1056/NEJMoa1606774 27718847

[B27] Ren S. Feng J. Ma S. Chen H. Ma Z. Huang C. (2023). KEYNOTE-033: randomized phase 3 study of pembrolizumab vs docetaxel in previously treated, PD-L1-positive, advanced NSCLC. Intl J. Cancer 153, 623–634. 10.1002/ijc.34532 37141294

[B28] Ribas A. Wolchok J. D. (2018). Cancer immunotherapy using checkpoint blockade. Science 359, 1350–1355. 10.1126/science.aar4060 29567705 PMC7391259

[B29] Yang S. (2019). Interim monitoring using the adaptively weighted log-rank test in clinical trials for survival outcomes: interim monitoring using the adaptively weighted log-rank test. Statistics Med. 38, 601–612. 10.1002/sim.7958 30209818

