# Supplementary material for: A change point-based analysis procedure for improving the success rate of decision-making in clinical trials with delayed treatment effects
Source: Front Pharmacol. 2023 Sep 11;14:1186456. doi: 10.3389/fphar.2023.1186456 (PMC10520459; doi:10.3389/fphar.2023.1186456)
Supplement: Supplementary file 1 [file DataSheet1.docx]

Supplementary Material

A change point-based analysis procedure for improving the success rate of decision-making in clinical trials with delayed treatment effects

Long-Shen Xie, Hui Lu*

*** Correspondence:** Hui Lu: huilu@sjtu.edu.cn

# Supplementary Figures and Tables

## Supplementary Figures


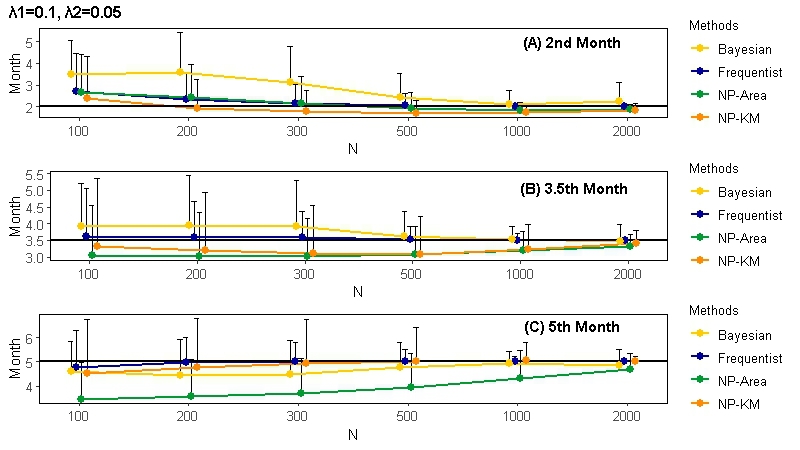


**Supplementary Figure S1.** Mean and SD of true change points and change points estimated by different methods under various scenarios for 1000 simulations. Panels A, B, and C represent change points occurring in the 2nd, 3.5th, and 5th months after the first administration, respectively. The black line represents the true value of the change point. The censoring rate is zero. SD is represented by the length of the whisker. The true hazard rates before and after the change point are 0.1 and 0.05, respectively. NP is non-parametric. KM is Kaplan-Meier.

## Supplementary Tables

**Supplementary Table S1.** The performance of different change point detection methods under various scenarios.

Sample size=200

| True CP | Methods | True hazard rate $\lambda_{1}$=0.1, $\lambda_{2}$=0.05 | | | True hazard rate $\lambda_{1}$=0.1, $\lambda_{2}$=0.067 | | | True hazard rate $\lambda_{1}$=0.1, $\lambda_{2}$=0.1 | | |
| --- | --- | --- | --- | --- | --- | --- | --- | --- | --- | --- |
|  |  | CR=0 | CR=20% | CR=50% | CR=0 | CR=20% | CR=50% | CR=0 | CR=20% | CR=50% |
| 2nd month | Profile likelihood | $\hat{CP}$=2.33  $\hat{\lambda}_{1}$=0.102  $\hat{\lambda}_{2}$=0.050 | $\hat{CP}$=2.31  $\hat{\lambda}_{1}$=0.102  $\hat{\lambda}_{2}$=0.050 | $\hat{CP}$=2.37  $\hat{\lambda}_{1}$=0.102  $\hat{\lambda}_{2}$=0.049 | $\hat{CP}$=2.86  $\hat{\lambda}_{1}$=0.103  $\hat{\lambda}_{2}$=0.066 | $\hat{CP}$=2.91  $\hat{\lambda}_{1}$=0.103  $\hat{\lambda}_{2}$=0.066 | $\hat{CP}$=3.05  $\hat{\lambda}_{1}$=0.102  $\hat{\lambda}_{2}$=0.065 | $\hat{CP}$=3.98  $\hat{\lambda}_{1}$=0.100  $\hat{\lambda}_{2}$=0.101 | $\hat{CP}$=4.08  $\hat{\lambda}_{1}$=0.100  $\hat{\lambda}_{2}$=0.102 | $\hat{CP}$=4.36  $\hat{\lambda}_{1}$=0.100  $\hat{\lambda}_{2}$=0.106 |
|  | Bayesian Exponential | $\hat{CP}$=3.59  $\hat{\lambda}_{1}$=0.088  $\hat{\lambda}_{2}$=0.050 | $\hat{CP}$=3.50  $\hat{\lambda}_{1}$=0.089  $\hat{\lambda}_{2}$=0.050 | $\hat{CP}$=3.30  $\hat{\lambda}_{1}$=0.092  $\hat{\lambda}_{2}$=0.050 | $\hat{CP}$=3.68  $\hat{\lambda}_{1}$=0.093  $\hat{\lambda}_{2}$=0.067 | $\hat{CP}$=3.64  $\hat{\lambda}_{1}$=0.094  $\hat{\lambda}_{2}$=0.067 | $\hat{CP}$=3.86  $\hat{\lambda}_{1}$=0.094  $\hat{\lambda}_{2}$=0.067 | $\hat{CP}$=4.19  $\hat{\lambda}_{1}$=0.100  $\hat{\lambda}_{2}$=0.101 | $\hat{CP}$=4.29  $\hat{\lambda}_{1}$=0.100  $\hat{\lambda}_{2}$=0.102 | $\hat{CP}$=4.74  $\hat{\lambda}_{1}$=0.100  $\hat{\lambda}_{2}$=0.103 |
|  | NP-Area | $\hat{CP}$=2.41  $\hat{\lambda}_{1}$=0.101  $\hat{\lambda}_{2}$=0.050 | $\hat{CP}$=2.41  $\hat{\lambda}_{1}$=0.101  $\hat{\lambda}_{2}$=0.050 | $\hat{CP}$=2.43  $\hat{\lambda}_{1}$=0.101  $\hat{\lambda}_{2}$=0.050 | $\hat{CP}$=2.95  $\hat{\lambda}_{1}$=0.102  $\hat{\lambda}_{2}$=0.067 | $\hat{CP}$=2.95  $\hat{\lambda}_{1}$=0.102  $\hat{\lambda}_{2}$=0.066 | $\hat{CP}$=3.26  $\hat{\lambda}_{1}$=0.100  $\hat{\lambda}_{2}$=0.065 | $\hat{CP}$=3.85  $\hat{\lambda}_{1}$=0.109  $\hat{\lambda}_{2}$=0.098 | $\hat{CP}$=3.85  $\hat{\lambda}_{1}$=0.109  $\hat{\lambda}_{2}$=0.098 | $\hat{CP}$=4.47  $\hat{\lambda}_{1}$=0.107  $\hat{\lambda}_{2}$=0.094 |
|  | NP-KM | $\hat{CP}$=1.93  $\hat{\lambda}_{1}$=0.107  $\hat{\lambda}_{2}$=0.050 | $\hat{CP}$=1.93  $\hat{\lambda}_{1}$=0.107  $\hat{\lambda}_{2}$=0.051 | $\hat{CP}$=2.01  $\hat{\lambda}_{1}$=0.106  $\hat{\lambda}_{2}$=0.051 | $\hat{CP}$=2.82  $\hat{\lambda}_{1}$=0.105  $\hat{\lambda}_{2}$=0.067 | $\hat{CP}$=2.82  $\hat{\lambda}_{1}$=0.105  $\hat{\lambda}_{2}$=0.066 | $\hat{CP}$=3.14  $\hat{\lambda}_{1}$=0.104  $\hat{\lambda}_{2}$=0.064 | $\hat{CP}$=4.94  $\hat{\lambda}_{1}$=0.110  $\hat{\lambda}_{2}$=0.097 | $\hat{CP}$=4.93  $\hat{\lambda}_{1}$=0.110  $\hat{\lambda}_{2}$=0.096 | $\hat{CP}$=5.35  $\hat{\lambda}_{1}$=0.108  $\hat{\lambda}_{2}$=0.088 |
| 3.5th month | Profile likelihood | $\hat{CP}$=3.60  $\hat{\lambda}_{1}$=0.103  $\hat{\lambda}_{2}$=0.050 | $\hat{CP}$=3.61  $\hat{\lambda}_{1}$=0.103  $\hat{\lambda}_{2}$=0.050 | $\hat{CP}$=3.59  $\hat{\lambda}_{1}$=0.103  $\hat{\lambda}_{2}$=0.049 | $\hat{CP}$=3.69  $\hat{\lambda}_{1}$=0.105  $\hat{\lambda}_{2}$=0.067 | $\hat{CP}$=3.71  $\hat{\lambda}_{1}$=0.105  $\hat{\lambda}_{2}$=0.066 | $\hat{CP}$=3.80  $\hat{\lambda}_{1}$=0.104  $\hat{\lambda}_{2}$=0.065 | $\hat{CP}$=3.99  $\hat{\lambda}_{1}$=0.100  $\hat{\lambda}_{2}$=0.101 | $\hat{CP}$=4.08  $\hat{\lambda}_{1}$=0.100  $\hat{\lambda}_{2}$=0.102 | $\hat{CP}$=4.39  $\hat{\lambda}_{1}$=0.100  $\hat{\lambda}_{2}$=0.104 |
|  | Bayesian Exponential | $\hat{CP}$=3.95  $\hat{\lambda}_{1}$=0.098  $\hat{\lambda}_{2}$=0.051 | $\hat{CP}$=3.93  $\hat{\lambda}_{1}$=0.098  $\hat{\lambda}_{2}$=0.051 | $\hat{CP}$=3.91  $\hat{\lambda}_{1}$=0.099  $\hat{\lambda}_{2}$=0.051 | $\hat{CP}$=4.02  $\hat{\lambda}_{1}$=0.099  $\hat{\lambda}_{2}$=0.068 | $\hat{CP}$=3.99  $\hat{\lambda}_{1}$=0.100  $\hat{\lambda}_{2}$=0.068 | $\hat{CP}$=4.29  $\hat{\lambda}_{1}$=0.099  $\hat{\lambda}_{2}$=0.068 | $\hat{CP}$=4.19  $\hat{\lambda}_{1}$=0.100  $\hat{\lambda}_{2}$=0.101 | $\hat{CP}$=4.30  $\hat{\lambda}_{1}$=0.100  $\hat{\lambda}_{2}$=0.102 | $\hat{CP}$=4.75  $\hat{\lambda}_{1}$=0.100  $\hat{\lambda}_{2}$=0.102 |
|  | NP-Area | $\hat{CP}$=3.03  $\hat{\lambda}_{1}$=0.106  $\hat{\lambda}_{2}$=0.051 | $\hat{CP}$=3.03  $\hat{\lambda}_{1}$=0.106  $\hat{\lambda}_{2}$=0.052 | $\hat{CP}$=3.16  $\hat{\lambda}_{1}$=0.105  $\hat{\lambda}_{2}$=0.053 | $\hat{CP}$=3.23  $\hat{\lambda}_{1}$=0.106  $\hat{\lambda}_{2}$=0.065 | $\hat{CP}$=3.23  $\hat{\lambda}_{1}$=0.106  $\hat{\lambda}_{2}$=0.068 | $\hat{CP}$=3.65  $\hat{\lambda}_{1}$=0.105  $\hat{\lambda}_{2}$=0.068 | $\hat{CP}$=3.82  $\hat{\lambda}_{1}$=0.109  $\hat{\lambda}_{2}$=0.098 | $\hat{CP}$=3.82  $\hat{\lambda}_{1}$=0.109  $\hat{\lambda}_{2}$=0.097 | $\hat{CP}$=4.50  $\hat{\lambda}_{1}$=0.108  $\hat{\lambda}_{2}$=0.093 |
|  | NP-KM | $\hat{CP}$=3.2  $\hat{\lambda}_{1}$=0.107  $\hat{\lambda}_{2}$=0.051 | $\hat{CP}$=3.2  $\hat{\lambda}_{1}$=0.107  $\hat{\lambda}_{2}$=0.051 | $\hat{CP}$=3.32  $\hat{\lambda}_{1}$=0.107  $\hat{\lambda}_{2}$=0.052 | $\hat{CP}$=3.84  $\hat{\lambda}_{1}$=0.106  $\hat{\lambda}_{2}$=0.067 | $\hat{CP}$=3.84  $\hat{\lambda}_{1}$=0.106  $\hat{\lambda}_{2}$=0.067 | $\hat{CP}$=4.15  $\hat{\lambda}_{1}$=0.105  $\hat{\lambda}_{2}$=0.064 | $\hat{CP}$=4.74  $\hat{\lambda}_{1}$=0.110  $\hat{\lambda}_{2}$=0.098 | $\hat{CP}$=4.74  $\hat{\lambda}_{1}$=0.110  $\hat{\lambda}_{2}$=0.096 | $\hat{CP}$=5.14  $\hat{\lambda}_{1}$=0.109  $\hat{\lambda}_{2}$=0.087 |
| 5th month | Profile likelihood | $\hat{CP}$=4.98  $\hat{\lambda}_{1}$=0.102  $\hat{\lambda}_{2}$=0.050 | $\hat{CP}$=4.95  $\hat{\lambda}_{1}$=0.103  $\hat{\lambda}_{2}$=0.050 | $\hat{CP}$=4.85  $\hat{\lambda}_{1}$=0.104  $\hat{\lambda}_{2}$=0.050 | $\hat{CP}$=4.75  $\hat{\lambda}_{1}$=0.105  $\hat{\lambda}_{2}$=0.067 | $\hat{CP}$=4.62  $\hat{\lambda}_{1}$=0.105  $\hat{\lambda}_{2}$=0.067 | $\hat{CP}$=4.58  $\hat{\lambda}_{1}$=0.104  $\hat{\lambda}_{2}$=0.066 | $\hat{CP}$=3.94  $\hat{\lambda}_{1}$=0.100  $\hat{\lambda}_{2}$=0.102 | $\hat{CP}$=4.06  $\hat{\lambda}_{1}$=0.100  $\hat{\lambda}_{2}$=0.102 | $\hat{CP}$=4.35  $\hat{\lambda}_{1}$=0.100  $\hat{\lambda}_{2}$=0.106 |
|  | Bayesian Exponential | $\hat{CP}$=4.44  $\hat{\lambda}_{1}$=0.101  $\hat{\lambda}_{2}$=0.052 | $\hat{CP}$=4.60  $\hat{\lambda}_{1}$=0.101  $\hat{\lambda}_{2}$=0.052 | $\hat{CP}$=4.96  $\hat{\lambda}_{1}$=0.101  $\hat{\lambda}_{2}$=0.052 | $\hat{CP}$=4.42  $\hat{\lambda}_{1}$=0.102  $\hat{\lambda}_{2}$=0.069 | $\hat{CP}$=4.55  $\hat{\lambda}_{1}$=0.102  $\hat{\lambda}_{2}$=0.069 | $\hat{CP}$=4.82  $\hat{\lambda}_{1}$=0.101  $\hat{\lambda}_{2}$=0.070 | $\hat{CP}$=4.19  $\hat{\lambda}_{1}$=0.100  $\hat{\lambda}_{2}$=0.101 | $\hat{CP}$=4.29  $\hat{\lambda}_{1}$=0.100  $\hat{\lambda}_{2}$=0.102 | $\hat{CP}$=4.71  $\hat{\lambda}_{1}$=0.100  $\hat{\lambda}_{2}$=0.103 |
|  | NP-Area | $\hat{CP}$=3.57  $\hat{\lambda}_{1}$=0.107  $\hat{\lambda}_{2}$=0.053 | $\hat{CP}$=3.57  $\hat{\lambda}_{1}$=0.107  $\hat{\lambda}_{2}$=0.054 | $\hat{CP}$=3.81  $\hat{\lambda}_{1}$=0.107  $\hat{\lambda}_{2}$=0.059 | $\hat{CP}$=3.66  $\hat{\lambda}_{1}$=0.107  $\hat{\lambda}_{2}$=0.069 | $\hat{CP}$=3.66  $\hat{\lambda}_{1}$=0.107  $\hat{\lambda}_{2}$=0.070 | $\hat{CP}$=3.95  $\hat{\lambda}_{1}$=0.107  $\hat{\lambda}_{2}$=0.071 | $\hat{CP}$=3.85  $\hat{\lambda}_{1}$=0.109  $\hat{\lambda}_{2}$=0.098 | $\hat{CP}$=3.86  $\hat{\lambda}_{1}$=0.109  $\hat{\lambda}_{2}$=0.098 | $\hat{CP}$=4.49  $\hat{\lambda}_{1}$=0.108  $\hat{\lambda}_{2}$=0.094 |
|  | NP-KM | $\hat{CP}$=4.77  $\hat{\lambda}_{1}$=0.105  $\hat{\lambda}_{2}$=0.051 | $\hat{CP}$=4.77  $\hat{\lambda}_{1}$=0.105  $\hat{\lambda}_{2}$=0.051 | $\hat{CP}$=4.92  $\hat{\lambda}_{1}$=0.105  $\hat{\lambda}_{2}$=0.051 | $\hat{CP}$=4.90  $\hat{\lambda}_{1}$=0.106  $\hat{\lambda}_{2}$=0.067 | $\hat{CP}$=4.90  $\hat{\lambda}_{1}$=0.106  $\hat{\lambda}_{2}$=0.067 | $\hat{CP}$=5.11  $\hat{\lambda}_{1}$=0.106  $\hat{\lambda}_{2}$=0.064 | $\hat{CP}$=4.84  $\hat{\lambda}_{1}$=0.110  $\hat{\lambda}_{2}$=0.098 | $\hat{CP}$=4.83  $\hat{\lambda}_{1}$=0.110  $\hat{\lambda}_{2}$=0.096 | $\hat{CP}$=5.25  $\hat{\lambda}_{1}$=0.109  $\hat{\lambda}_{2}$=0.087 |

Note: CP is the abbreviation of change point. $\lambda_{1}$ and $\lambda_{2}$ are true hazard rates before and after the change point. $\hat{CP}$, $\hat{\lambda}_{1}$ and $\hat{\lambda}_{2}$ are estimates of change point, $\lambda_{1}$ and $\lambda_{2}$, respectively. CR means censoring rate. NP is non-parametric. KM is Kaplan-Meier.

Sample size =300

| True CP | Methods | True hazard rate $\lambda_{1}$=0.1, $\lambda_{2}$=0.05 | | | True hazard rate $\lambda_{1}$=0.1, $\lambda_{2}$=0.067 | | | True hazard rate $\lambda_{1}$=0.1, $\lambda_{2}$=0.1 | | |
| --- | --- | --- | --- | --- | --- | --- | --- | --- | --- | --- |
|  |  | CR=0 | CR=20% | CR=50% | CR=0 | CR=20% | CR=50% | CR=0 | CR=20% | CR=50% |
| 2nd month | Profile likelihood | $\hat{CP}$=2.17  $\hat{\lambda}_{1}$=0.101  $\hat{\lambda}_{2}$=0.050 | $\hat{CP}$=2.18  $\hat{\lambda}_{1}$=0.101  $\hat{\lambda}_{2}$=0.050 | $\hat{CP}$=2.18  $\hat{\lambda}_{1}$=0.101  $\hat{\lambda}_{2}$=0.050 | $\hat{CP}$=2.61  $\hat{\lambda}_{1}$=0.102  $\hat{\lambda}_{2}$=0.066 | $\hat{CP}$=2.66  $\hat{\lambda}_{1}$=0.101  $\hat{\lambda}_{2}$=0.066 | $\hat{CP}$=2.76  $\hat{\lambda}_{1}$=0.101  $\hat{\lambda}_{2}$=0.065 | $\hat{CP}$=4.13  $\hat{\lambda}_{1}$=0.099  $\hat{\lambda}_{2}$=0.101 | $\hat{CP}$=4.21  $\hat{\lambda}_{1}$=0.099  $\hat{\lambda}_{2}$=0.102 | $\hat{CP}$=4.46  $\hat{\lambda}_{1}$=0.099  $\hat{\lambda}_{2}$=0.103 |
|  | Bayesian Exponential | $\hat{CP}$=3.14  $\hat{\lambda}_{1}$=0.091  $\hat{\lambda}_{2}$=0.050 | $\hat{CP}$=3.50  $\hat{\lambda}_{1}$=0.088  $\hat{\lambda}_{2}$=0.050 | $\hat{CP}$=3.53  $\hat{\lambda}_{1}$=0.089  $\hat{\lambda}_{2}$=0.050 | $\hat{CP}$=3.77  $\hat{\lambda}_{1}$=0.091  $\hat{\lambda}_{2}$=0.067 | $\hat{CP}$=3.68  $\hat{\lambda}_{1}$=0.092  $\hat{\lambda}_{2}$=0.067 | $\hat{CP}$=3.76  $\hat{\lambda}_{1}$=0.093  $\hat{\lambda}_{2}$=0.066 | $\hat{CP}$=4.20  $\hat{\lambda}_{1}$=0.100  $\hat{\lambda}_{2}$=0.101 | $\hat{CP}$=4.28  $\hat{\lambda}_{1}$=0.100  $\hat{\lambda}_{2}$=0.101 | $\hat{CP}$=4.74  $\hat{\lambda}_{1}$=0.100  $\hat{\lambda}_{2}$=0.102 |
|  | NP-Area | $\hat{CP}$=2.16  $\hat{\lambda}_{1}$=0.101  $\hat{\lambda}_{2}$=0.050 | $\hat{CP}$=2.16  $\hat{\lambda}_{1}$=0.101  $\hat{\lambda}_{2}$=0.050 | $\hat{CP}$=2.20  $\hat{\lambda}_{1}$=0.101  $\hat{\lambda}_{2}$=0.051 | $\hat{CP}$=2.61  $\hat{\lambda}_{1}$=0.101  $\hat{\lambda}_{2}$=0.067 | $\hat{CP}$=2.61  $\hat{\lambda}_{1}$=0.101  $\hat{\lambda}_{2}$=0.067 | $\hat{CP}$=2.94  $\hat{\lambda}_{1}$=0.100  $\hat{\lambda}_{2}$=0.066 | $\hat{CP}$=3.78  $\hat{\lambda}_{1}$=0.106  $\hat{\lambda}_{2}$=0.099 | $\hat{CP}$=3.78  $\hat{\lambda}_{1}$=0.106  $\hat{\lambda}_{2}$=0.098 | $\hat{CP}$=4.53  $\hat{\lambda}_{1}$=0.106  $\hat{\lambda}_{2}$=0.094 |
|  | NP-KM | $\hat{CP}$=1.78  $\hat{\lambda}_{1}$=0.105  $\hat{\lambda}_{2}$=0.051 | $\hat{CP}$=1.78  $\hat{\lambda}_{1}$=0.105  $\hat{\lambda}_{2}$=0.051 | $\hat{CP}$=1.79  $\hat{\lambda}_{1}$=0.105  $\hat{\lambda}_{2}$=0.051 | $\hat{CP}$=2.45  $\hat{\lambda}_{1}$=0.104  $\hat{\lambda}_{2}$=0.067 | $\hat{CP}$=2.45  $\hat{\lambda}_{1}$=0.104  $\hat{\lambda}_{2}$=0.067 | $\hat{CP}$=2.67  $\hat{\lambda}_{1}$=0.103  $\hat{\lambda}_{2}$=0.066 | $\hat{CP}$=4.89  $\hat{\lambda}_{1}$=0.108  $\hat{\lambda}_{2}$=0.098 | $\hat{CP}$=4.88  $\hat{\lambda}_{1}$=0.108  $\hat{\lambda}_{2}$=0.097 | $\hat{CP}$=5.36  $\hat{\lambda}_{1}$=0.106  $\hat{\lambda}_{2}$=0.090 |
| 3.5th months | Profile likelihood | $\hat{CP}$=3.58  $\hat{\lambda}_{1}$=0.101  $\hat{\lambda}_{2}$=0.050 | $\hat{CP}$=3.58  $\hat{\lambda}_{1}$=0.101  $\hat{\lambda}_{2}$=0.050 | $\hat{CP}$=3.55  $\hat{\lambda}_{1}$=0.102  $\hat{\lambda}_{2}$=0.050 | $\hat{CP}$=3.64  $\hat{\lambda}_{1}$=0.103  $\hat{\lambda}_{2}$=0.067 | $\hat{CP}$=3.69  $\hat{\lambda}_{1}$=0.103  $\hat{\lambda}_{2}$=0.066 | $\hat{CP}$=3.75  $\hat{\lambda}_{1}$=0.103  $\hat{\lambda}_{2}$=0.065 | $\hat{CP}$=4.12  $\hat{\lambda}_{1}$=0.100  $\hat{\lambda}_{2}$=0.101 | $\hat{CP}$=4.15  $\hat{\lambda}_{1}$=0.099  $\hat{\lambda}_{2}$=0.102 | $\hat{CP}$=4.47  $\hat{\lambda}_{1}$=0.100  $\hat{\lambda}_{2}$=0.103 |
|  | Bayesian Exponential | $\hat{CP}$=3.90  $\hat{\lambda}_{1}$=0.098  $\hat{\lambda}_{2}$=0.051 | $\hat{CP}$=3.97  $\hat{\lambda}_{1}$=0.097  $\hat{\lambda}_{2}$=0.051 | $\hat{CP}$=4.01  $\hat{\lambda}_{1}$=0.098  $\hat{\lambda}_{2}$=0.051 | $\hat{CP}$=4.07  $\hat{\lambda}_{1}$=0.098  $\hat{\lambda}_{2}$=0.068 | $\hat{CP}$=4.05  $\hat{\lambda}_{1}$=0.098  $\hat{\lambda}_{2}$=0.068 | $\hat{CP}$=4.25  $\hat{\lambda}_{1}$=0.099  $\hat{\lambda}_{2}$=0.067 | $\hat{CP}$=4.22  $\hat{\lambda}_{1}$=0.100  $\hat{\lambda}_{2}$=0.101 | $\hat{CP}$=4.29  $\hat{\lambda}_{1}$=0.100  $\hat{\lambda}_{2}$=0.101 | $\hat{CP}$=4.74  $\hat{\lambda}_{1}$=0.100  $\hat{\lambda}_{2}$=0.101 |
|  | NP-Area | $\hat{CP}$=3.02  $\hat{\lambda}_{1}$=0.104  $\hat{\lambda}_{2}$=0.051 | $\hat{CP}$=3.02  $\hat{\lambda}_{1}$=0.104  $\hat{\lambda}_{2}$=0.052 | $\hat{CP}$=3.10  $\hat{\lambda}_{1}$=0.104  $\hat{\lambda}_{2}$=0.053 | $\hat{CP}$=3.09  $\hat{\lambda}_{1}$=0.104  $\hat{\lambda}_{2}$=0.068 | $\hat{CP}$=3.09  $\hat{\lambda}_{1}$=0.104  $\hat{\lambda}_{2}$=0.068 | $\hat{CP}$=3.44  $\hat{\lambda}_{1}$=0.103  $\hat{\lambda}_{2}$=0.068 | $\hat{CP}$=3.82  $\hat{\lambda}_{1}$=0.106  $\hat{\lambda}_{2}$=0.099 | $\hat{CP}$=3.82  $\hat{\lambda}_{1}$=0.106  $\hat{\lambda}_{2}$=0.098 | $\hat{CP}$=4.46  $\hat{\lambda}_{1}$=0.106  $\hat{\lambda}_{2}$=0.093 |
|  | NP-KM | $\hat{CP}$=3.10  $\hat{\lambda}_{1}$=0.105  $\hat{\lambda}_{2}$=0.051 | $\hat{CP}$=3.10  $\hat{\lambda}_{1}$=0.105  $\hat{\lambda}_{2}$=0.051 | $\hat{CP}$=3.22  $\hat{\lambda}_{1}$=0.105  $\hat{\lambda}_{2}$=0.052 | $\hat{CP}$=3.51  $\hat{\lambda}_{1}$=0.105  $\hat{\lambda}_{2}$=0.067 | $\hat{CP}$=3.51  $\hat{\lambda}_{1}$=0.105  $\hat{\lambda}_{2}$=0.067 | $\hat{CP}$=4.01  $\hat{\lambda}_{1}$=0.104  $\hat{\lambda}_{2}$=0.065 | $\hat{CP}$=4.72  $\hat{\lambda}_{1}$=0.108  $\hat{\lambda}_{2}$=0.098 | $\hat{CP}$=4.72  $\hat{\lambda}_{1}$=0.108  $\hat{\lambda}_{2}$=0.096 | $\hat{CP}$=5.24  $\hat{\lambda}_{1}$=0.107  $\hat{\lambda}_{2}$=0.089 |
| 5th month | Profile likelihood | $\hat{CP}$=5.02  $\hat{\lambda}_{1}$=0.101  $\hat{\lambda}_{2}$=0.050 | $\hat{CP}$=5.00  $\hat{\lambda}_{1}$=0.101  $\hat{\lambda}_{2}$=0.050 | $\hat{CP}$=4.95  $\hat{\lambda}_{1}$=0.102  $\hat{\lambda}_{2}$=0.050 | $\hat{CP}$=4.89  $\hat{\lambda}_{1}$=0.103  $\hat{\lambda}_{2}$=0.067 | $\hat{CP}$=4.84  $\hat{\lambda}_{1}$=0.103  $\hat{\lambda}_{2}$=0.067 | $\hat{CP}$=4.69  $\hat{\lambda}_{1}$=0.103  $\hat{\lambda}_{2}$=0.066 | $\hat{CP}$=4.02  $\hat{\lambda}_{1}$=0.100  $\hat{\lambda}_{2}$=0.101 | $\hat{CP}$=4.15  $\hat{\lambda}_{1}$=0.099  $\hat{\lambda}_{2}$=0.102 | $\hat{CP}$=4.37  $\hat{\lambda}_{1}$=0.100  $\hat{\lambda}_{2}$=0.103 |
|  | Bayesian Exponential | $\hat{CP}$=4.48  $\hat{\lambda}_{1}$=0.101  $\hat{\lambda}_{2}$=0.052 | $\hat{CP}$=4.50  $\hat{\lambda}_{1}$=0.101  $\hat{\lambda}_{2}$=0.053 | $\hat{CP}$=4.95  $\hat{\lambda}_{1}$=0.100  $\hat{\lambda}_{2}$=0.052 | $\hat{CP}$=4.44  $\hat{\lambda}_{1}$=0.101  $\hat{\lambda}_{2}$=0.069 | $\hat{CP}$=4.46  $\hat{\lambda}_{1}$=0.101  $\hat{\lambda}_{2}$=0.069 | $\hat{CP}$=4.89  $\hat{\lambda}_{1}$=0.101  $\hat{\lambda}_{2}$=0.069 | $\hat{CP}$=4.22  $\hat{\lambda}_{1}$=0.100  $\hat{\lambda}_{2}$=0.101 | $\hat{CP}$=4.33  $\hat{\lambda}_{1}$=0.100  $\hat{\lambda}_{2}$=0.101 | $\hat{CP}$=4.73  $\hat{\lambda}_{1}$=0.100  $\hat{\lambda}_{2}$=0.102 |
|  | NP-Area | $\hat{CP}$=3.68  $\hat{\lambda}_{1}$=0.105  $\hat{\lambda}_{2}$=0.053 | $\hat{CP}$=3.68  $\hat{\lambda}_{1}$=0.105  $\hat{\lambda}_{2}$=0.054 | $\hat{CP}$=3.83  $\hat{\lambda}_{1}$=0.105  $\hat{\lambda}_{2}$=0.058 | $\hat{CP}$=3.59  $\hat{\lambda}_{1}$=0.106  $\hat{\lambda}_{2}$=0.070 | $\hat{CP}$=3.59  $\hat{\lambda}_{1}$=0.106  $\hat{\lambda}_{2}$=0.070 | $\hat{CP}$=4.00  $\hat{\lambda}_{1}$=0.105  $\hat{\lambda}_{2}$=0.071 | $\hat{CP}$=3.79  $\hat{\lambda}_{1}$=0.106  $\hat{\lambda}_{2}$=0.099 | $\hat{CP}$=3.79  $\hat{\lambda}_{1}$=0.106  $\hat{\lambda}_{2}$=0.098 | $\hat{CP}$=4.49  $\hat{\lambda}_{1}$=0.106  $\hat{\lambda}_{2}$=0.094 |
|  | NP-KM | $\hat{CP}$=4.93  $\hat{\lambda}_{1}$=0.103  $\hat{\lambda}_{2}$=0.051 | $\hat{CP}$=4.93  $\hat{\lambda}_{1}$=0.103  $\hat{\lambda}_{2}$=0.051 | $\hat{CP}$=5.11  $\hat{\lambda}_{1}$=0.102  $\hat{\lambda}_{2}$=0.050 | $\hat{CP}$=4.97  $\hat{\lambda}_{1}$=0.104  $\hat{\lambda}_{2}$=0.067 | $\hat{CP}$=4.97  $\hat{\lambda}_{1}$=0.104  $\hat{\lambda}_{2}$=0.067 | $\hat{CP}$=5.20  $\hat{\lambda}_{1}$=0.104  $\hat{\lambda}_{2}$=0.065 | $\hat{CP}$=4.81  $\hat{\lambda}_{1}$=0.108  $\hat{\lambda}_{2}$=0.098 | $\hat{CP}$=4.81  $\hat{\lambda}_{1}$=0.108  $\hat{\lambda}_{2}$=0.096 | $\hat{CP}$=5.25  $\hat{\lambda}_{1}$=0.107  $\hat{\lambda}_{2}$=0.089 |

Note: CP is the abbreviation of change point. $\lambda_{1}$ and $\lambda_{2}$ are true hazard rates before and after the change point. $\hat{CP}$, $\hat{\lambda}_{1}$ and $\hat{\lambda}_{2}$ are estimates of change point, $\lambda_{1}$ and $\lambda_{2}$, respectively. CR means censoring rate. NP is non-parametric. KM is Kaplan-Meier.

Sample size =500

| True CP | Methods | True hazard rate $\lambda_{1}$=0.1, $\lambda_{2}$=0.05 | | | True hazard rate $\lambda_{1}$=0.1, $\lambda_{2}$=0.067 | | | True hazard rate $\lambda_{1}$=0.1, $\lambda_{2}$=0.1 | | |
| --- | --- | --- | --- | --- | --- | --- | --- | --- | --- | --- |
|  |  | CR=0 | CR=20% | CR=50% | CR=0 | CR=20% | CR=50% | CR=0 | CR=20% | CR=50% |
| 2nd month | Profile likelihood | $\hat{CP}$=2.1  $\hat{\lambda}_{1}$=0.100  $\hat{\lambda}_{2}$=0.050 | $\hat{CP}$=2.1  $\hat{\lambda}_{1}$=0.100  $\hat{\lambda}_{2}$=0.050 | $\hat{CP}$=2.1  $\hat{\lambda}_{1}$=0.100  $\hat{\lambda}_{2}$=0.050 | $\hat{CP}$=2.3  $\hat{\lambda}_{1}$=0.101  $\hat{\lambda}_{2}$=0.067 | $\hat{CP}$=2.3  $\hat{\lambda}_{1}$=0.101  $\hat{\lambda}_{2}$=0.067 | $\hat{CP}$=2.4  $\hat{\lambda}_{1}$=0.101  $\hat{\lambda}_{2}$=0.066 | $\hat{CP}$=4.1  $\hat{\lambda}_{1}$=0.099  $\hat{\lambda}_{2}$=0.101 | $\hat{CP}$=4.2  $\hat{\lambda}_{1}$=0.099  $\hat{\lambda}_{2}$=0.101 | $\hat{CP}$=4.2  $\hat{\lambda}_{1}$=0.099  $\hat{\lambda}_{2}$=0.101 |
|  | Bayesian Exponential | $\hat{CP}$=2.4  $\hat{\lambda}_{1}$=0.097  $\hat{\lambda}_{2}$=0.050 | $\hat{CP}$=2.7  $\hat{\lambda}_{1}$=0.095  $\hat{\lambda}_{2}$=0.050 | $\hat{CP}$=3.7  $\hat{\lambda}_{1}$=0.087  $\hat{\lambda}_{2}$=0.050 | $\hat{CP}$=3.4  $\hat{\lambda}_{1}$=0.093  $\hat{\lambda}_{2}$=0.067 | $\hat{CP}$=3.8  $\hat{\lambda}_{1}$=0.090  $\hat{\lambda}_{2}$=0.067 | $\hat{CP}$=4.0  $\hat{\lambda}_{1}$=0.090  $\hat{\lambda}_{2}$=0.067 | $\hat{CP}$=4.2  $\hat{\lambda}_{1}$=0.100  $\hat{\lambda}_{2}$=0.100 | $\hat{CP}$=4.3  $\hat{\lambda}_{1}$=0.100  $\hat{\lambda}_{2}$=0.101 | $\hat{CP}$=4.7  $\hat{\lambda}_{1}$=0.100  $\hat{\lambda}_{2}$=0.101 |
|  | NP-Area | $\hat{CP}$=1.91  $\hat{\lambda}_{1}$=0.102  $\hat{\lambda}_{2}$=0.050 | $\hat{CP}$=1.91  $\hat{\lambda}_{1}$=0.102  $\hat{\lambda}_{2}$=0.050 | $\hat{CP}$=1.90  $\hat{\lambda}_{1}$=0.102  $\hat{\lambda}_{2}$=0.051 | $\hat{CP}$=2.30  $\hat{\lambda}_{1}$=0.101  $\hat{\lambda}_{2}$=0.067 | $\hat{CP}$=2.30  $\hat{\lambda}_{1}$=0.101  $\hat{\lambda}_{2}$=0.067 | $\hat{CP}$=2.59  $\hat{\lambda}_{1}$=0.100  $\hat{\lambda}_{2}$=0.066 | $\hat{CP}$=3.70  $\hat{\lambda}_{1}$=0.105  $\hat{\lambda}_{2}$=0.099 | $\hat{CP}$=3.70  $\hat{\lambda}_{1}$=0.105  $\hat{\lambda}_{2}$=0.098 | $\hat{CP}$=4.47  $\hat{\lambda}_{1}$=0.104  $\hat{\lambda}_{2}$=0.095 |
|  | NP-KM | $\hat{CP}$=1.69  $\hat{\lambda}_{1}$=0.104  $\hat{\lambda}_{2}$=0.051 | $\hat{CP}$=1.69  $\hat{\lambda}_{1}$=0.104  $\hat{\lambda}_{2}$=0.051 | $\hat{CP}$=1.72  $\hat{\lambda}_{1}$=0.104  $\hat{\lambda}_{2}$=0.051 | $\hat{CP}$=2.08  $\hat{\lambda}_{1}$=0.103  $\hat{\lambda}_{2}$=0.067 | $\hat{CP}$=2.08  $\hat{\lambda}_{1}$=0.103  $\hat{\lambda}_{2}$=0.067 | $\hat{CP}$=2.35  $\hat{\lambda}_{1}$=0.103  $\hat{\lambda}_{2}$=0.067 | $\hat{CP}$=4.90  $\hat{\lambda}_{1}$=0.106  $\hat{\lambda}_{2}$=0.098 | $\hat{CP}$=4.90  $\hat{\lambda}_{1}$=0.106  $\hat{\lambda}_{2}$=0.097 | $\hat{CP}$=5.29  $\hat{\lambda}_{1}$=0.105  $\hat{\lambda}_{2}$=0.092 |
| 3.5th month | Profile likelihood | $\hat{CP}$=3.5  $\hat{\lambda}_{1}$=0.100  $\hat{\lambda}_{2}$=0.050 | $\hat{CP}$=3.5  $\hat{\lambda}_{1}$=0.100  $\hat{\lambda}_{2}$=0.050 | $\hat{CP}$=3.5  $\hat{\lambda}_{1}$=0.101  $\hat{\lambda}_{2}$=0.050 | $\hat{CP}$=3.6  $\hat{\lambda}_{1}$=0.101  $\hat{\lambda}_{2}$=0.067 | $\hat{CP}$=3.6  $\hat{\lambda}_{1}$=0.101  $\hat{\lambda}_{2}$=0.067 | $\hat{CP}$=3.6  $\hat{\lambda}_{1}$=0.102  $\hat{\lambda}_{2}$=0.066 | $\hat{CP}$=4.0  $\hat{\lambda}_{1}$=0.099  $\hat{\lambda}_{2}$=0.101 | $\hat{CP}$=4.1  $\hat{\lambda}_{1}$=0.099  $\hat{\lambda}_{2}$=0.101 | $\hat{CP}$=4.2  $\hat{\lambda}_{1}$=0.100  $\hat{\lambda}_{2}$=0.101 |
|  | Bayesian Exponential | $\hat{CP}$=3.6  $\hat{\lambda}_{1}$=0.099  $\hat{\lambda}_{2}$=0.050 | $\hat{CP}$=3.7  $\hat{\lambda}_{1}$=0.098  $\hat{\lambda}_{2}$=0.051 | $\hat{CP}$=4.1  $\hat{\lambda}_{1}$=0.096  $\hat{\lambda}_{2}$=0.051 | $\hat{CP}$=4.0  $\hat{\lambda}_{1}$=0.098  $\hat{\lambda}_{2}$=0.068 | $\hat{CP}$=4.1  $\hat{\lambda}_{1}$=0.097  $\hat{\lambda}_{2}$=0.068 | $\hat{CP}$=4.3  $\hat{\lambda}_{1}$=0.097  $\hat{\lambda}_{2}$=0.067 | $\hat{CP}$=4.2  $\hat{\lambda}_{1}$=0.100  $\hat{\lambda}_{2}$=0.101 | $\hat{CP}$=4.3  $\hat{\lambda}_{1}$=0.100  $\hat{\lambda}_{2}$=0.101 | $\hat{CP}$=4.7  $\hat{\lambda}_{1}$=0.100  $\hat{\lambda}_{2}$=0.101 |
|  | NP-Area | $\hat{CP}$=3.07  $\hat{\lambda}_{1}$=0.103  $\hat{\lambda}_{2}$=0.051 | $\hat{CP}$=3.07  $\hat{\lambda}_{1}$=0.103  $\hat{\lambda}_{2}$=0.051 | $\hat{CP}$=3.12  $\hat{\lambda}_{1}$=0.102  $\hat{\lambda}_{2}$=0.052 | $\hat{CP}$=3.03  $\hat{\lambda}_{1}$=0.103  $\hat{\lambda}_{2}$=0.068 | $\hat{CP}$=3.03  $\hat{\lambda}_{1}$=0.103  $\hat{\lambda}_{2}$=0.068 | $\hat{CP}$=3.34  $\hat{\lambda}_{1}$=0.102  $\hat{\lambda}_{2}$=0.068 | $\hat{CP}$=3.67  $\hat{\lambda}_{1}$=0.105  $\hat{\lambda}_{2}$=0.099 | $\hat{CP}$=3.67  $\hat{\lambda}_{1}$=0.105  $\hat{\lambda}_{2}$=0.098 | $\hat{CP}$=4.40  $\hat{\lambda}_{1}$=0.104  $\hat{\lambda}_{2}$=0.095 |
|  | NP-KM | $\hat{CP}$=3.09  $\hat{\lambda}_{1}$=0.104  $\hat{\lambda}_{2}$=0.051 | $\hat{CP}$=3.09  $\hat{\lambda}_{1}$=0.104  $\hat{\lambda}_{2}$=0.051 | $\hat{CP}$=3.10  $\hat{\lambda}_{1}$=0.104  $\hat{\lambda}_{2}$=0.052 | $\hat{CP}$=3.49  $\hat{\lambda}_{1}$=0.103  $\hat{\lambda}_{2}$=0.068 | $\hat{CP}$=3.49  $\hat{\lambda}_{1}$=0.103  $\hat{\lambda}_{2}$=0.068 | $\hat{CP}$=3.82  $\hat{\lambda}_{1}$=0.102  $\hat{\lambda}_{2}$=0.066 | $\hat{CP}$=4.70  $\hat{\lambda}_{1}$=0.106  $\hat{\lambda}_{2}$=0.098 | $\hat{CP}$=4.71  $\hat{\lambda}_{1}$=0.106  $\hat{\lambda}_{2}$=0.097 | $\hat{CP}$=5.12  $\hat{\lambda}_{1}$=0.105  $\hat{\lambda}_{2}$=0.091 |
| 5th month | Profile likelihood | $\hat{CP}$=5.0  $\hat{\lambda}_{1}$=0.100  $\hat{\lambda}_{2}$=0.050 | $\hat{CP}$=5.0  $\hat{\lambda}_{1}$=0.101  $\hat{\lambda}_{2}$=0.050 | $\hat{CP}$=5.0  $\hat{\lambda}_{1}$=0.101  $\hat{\lambda}_{2}$=0.050 | $\hat{CP}$=5.0  $\hat{\lambda}_{1}$=0.102  $\hat{\lambda}_{2}$=0.067 | $\hat{CP}$=5.0  $\hat{\lambda}_{1}$=0.102  $\hat{\lambda}_{2}$=0.067 | $\hat{CP}$=4.9  $\hat{\lambda}_{1}$=0.102  $\hat{\lambda}_{2}$=0.065 | $\hat{CP}$=4.0  $\hat{\lambda}_{1}$=0.099  $\hat{\lambda}_{2}$=0.101 | $\hat{CP}$=4.0  $\hat{\lambda}_{1}$=0.099  $\hat{\lambda}_{2}$=0.101 | $\hat{CP}$=4.3  $\hat{\lambda}_{1}$=0.100  $\hat{\lambda}_{2}$=0.101 |
|  | Bayesian Exponential | $\hat{CP}$= 4.8  $\hat{\lambda}_{1}$=0.100  $\hat{\lambda}_{2}$=0.051 | $\hat{CP}$=4.5  $\hat{\lambda}_{1}$=0.100  $\hat{\lambda}_{2}$=0.052 | $\hat{CP}$=4.8  $\hat{\lambda}_{1}$=0.100  $\hat{\lambda}_{2}$=0.053 | $\hat{CP}$=4.3  $\hat{\lambda}_{1}$=0.101  $\hat{\lambda}_{2}$=0.069 | $\hat{CP}$=4.5  $\hat{\lambda}_{1}$=0.100  $\hat{\lambda}_{2}$=0.069 | $\hat{CP}$=4.8  $\hat{\lambda}_{1}$=0.100  $\hat{\lambda}_{2}$=0.069 | $\hat{CP}$=4.2  $\hat{\lambda}_{1}$=0.100  $\hat{\lambda}_{2}$=0.101 | $\hat{CP}$=4.3  $\hat{\lambda}_{1}$=0.100  $\hat{\lambda}_{2}$=0.101 | $\hat{CP}$=4.7  $\hat{\lambda}_{1}$=0.100  $\hat{\lambda}_{2}$=0.101 |
|  | NP-Area | $\hat{CP}$=3.95  $\hat{\lambda}_{1}$=0.104  $\hat{\lambda}_{2}$=0.052 | $\hat{CP}$=3.95  $\hat{\lambda}_{1}$=0.104  $\hat{\lambda}_{2}$=0.053 | $\hat{CP}$=4.04  $\hat{\lambda}_{1}$=0.103  $\hat{\lambda}_{2}$=0.057 | $\hat{CP}$=3.71  $\hat{\lambda}_{1}$=0.104  $\hat{\lambda}_{2}$=0.069 | $\hat{CP}$=3.71  $\hat{\lambda}_{1}$=0.104  $\hat{\lambda}_{2}$=0.070 | $\hat{CP}$=4.03  $\hat{\lambda}_{1}$=0.104  $\hat{\lambda}_{2}$=0.071 | $\hat{CP}$=3.72  $\hat{\lambda}_{1}$=0.105  $\hat{\lambda}_{2}$=0.099 | $\hat{CP}$=3.72  $\hat{\lambda}_{1}$=0.105  $\hat{\lambda}_{2}$=0.098 | $\hat{CP}$=4.45  $\hat{\lambda}_{1}$=0.105  $\hat{\lambda}_{2}$=0.094 |
|  | NP-KM | $\hat{CP}$=5.02  $\hat{\lambda}_{1}$=0.101  $\hat{\lambda}_{2}$=0.050 | $\hat{CP}$=5.02  $\hat{\lambda}_{1}$=0.101  $\hat{\lambda}_{2}$=0.051 | $\hat{CP}$=5.20  $\hat{\lambda}_{1}$=0.101  $\hat{\lambda}_{2}$=0.050 | $\hat{CP}$=5.09  $\hat{\lambda}_{1}$=0.102  $\hat{\lambda}_{2}$=0.067 | $\hat{CP}$=5.09  $\hat{\lambda}_{1}$=0.102  $\hat{\lambda}_{2}$=0.067 | $\hat{CP}$=5.30  $\hat{\lambda}_{1}$=0.102  $\hat{\lambda}_{2}$=0.065 | $\hat{CP}$=4.89  $\hat{\lambda}_{1}$=0.106  $\hat{\lambda}_{2}$=0.098 | $\hat{CP}$=4.89  $\hat{\lambda}_{1}$=0.106  $\hat{\lambda}_{2}$=0.097 | $\hat{CP}$=5.28  $\hat{\lambda}_{1}$=0.105  $\hat{\lambda}_{2}$=0.091 |

Note: CP is the abbreviation of change point. $\lambda_{1}$ and $\lambda_{2}$ are true hazard rates before and after the change point. $\hat{CP}$, $\hat{\lambda}_{1}$ and $\hat{\lambda}_{2}$ are estimates of change point, $\lambda_{1}$ and $\lambda_{2}$, respectively. CR means censoring rate. NP is non-parametric. KM is Kaplan-Meier.

**Supplementary Table S2.** The percentage of significant change point tested by sequential likelihood ratio method in different groups under various conditions, and the percentage of non-proportional hazards tested by Cox regression.

Sample size=300

| Change point | True hazard rate: $\lambda_{T1}$=0.1, $\lambda_{T2}$=0.05, $\lambda_{C}$=0.1 | | | True hazard rate: $\lambda_{T1}$=0.1, $\lambda_{T2}$=0.067, $\lambda_{C}$=0.1 | | | True hazard rate: $\lambda_{T1}$=0.1, $\lambda_{T2}$=0.1, $\lambda_{C}$=0.1 | | | |
| --- | --- | --- | --- | --- | --- | --- | --- | --- | --- | --- |
|  | CR=0 | CR=20% | CR=50% | CR=0 | CR=20% | CR=50% | CR=0 | CR=20% | CR=50% |  |
| 2nd month | $T$=100%  $C$=3.9%  $P$=100%  $Cox$=44.8% | $T$=100%  $C$=4.5%  $P$=100%  $Cox$=42.8% | $T$=100%  $C$=3.6%  $P$=91.3%  $Cox$=35.4% | $T$=100%  $C$=3.9%  $P$=96.3%  $Cox$=17.0% | $T$=99.95  $C$=4.5%  $P$=86.0%  $Cox$=18.4% | $T$=98.1%  $C$=3.6%  $P$=54.0%  $Cox$=14.5% | $T$=3.0%  $C$=2.8%  $P$=2.8%  $Cox$=4.3% | $T$=6.9%  $C$=6.4%  $P$=6%  $Cox$=4.8% | $T$=5.1%  $C$=4.8%  $P$=5.8%  $Cox$=4.1% |  |
| 3.5th month | $T$=100%  $C$=4.5%  $P$=100%  $Cox$=59.7% | $T$=100%  $C$=5.2%  $P$=99.1%  $Cox$=52.7% | $T$=99.8%  $C$=4.1%  $P$=63.4%  $Cox$=29.8% | $T$=100%  $C$=4.5%  $P$=92.5%  $Cox$=24.8% | $T$=99.8%  $C$=5.2%  $P$=74.7%  $Cox$=20.4% | $T$=83.0%  $C$=4.1%  $P$=31.5%  $Cox$=12.2% | $T$=2.9%  $C$=3.7%  $P$=4.0%  $Cox$=4.5% | $T$=3.8%  $C$=4.1%  $P$=4.0%  $Cox$=4.8% | $T$=3.3%  $C$=4.6%  $P$=4.3%  $Cox$=4.6% |  |
| 5th month | $T$=100%  $C$=4.3%  $P$=100%  $Cox$=63.7% | $T$=100%  $C$=5.1%  $P$=95.6%  $Cox$=51.9% | $T$=95%  $C$=4.2%  $P$=43.1%  $Cox$=17.9% | $T$=100%  $C$=4.3%  $P$=87.6%  $Cox$=25.8% | $T$=98.8%  $C$=5.1%  $P$=57.7%  $Cox$=20.4% | $T$=62.6%  $C$=4.2%  $P$=20.4%  $Cox$=9.3% | $T$=3.1%  $C$=3.2%  $P$=3.6%  $Cox$=4.5% | $T$=4.7%  $C$=4.3%  $P$=4.3%  $Cox$=4.6% | $T$=4.4%  $C$=5.6%  $P$=5.6%  $Cox$=4.6% |  |

Note: $\lambda_{T1}$ and $\lambda_{T2}$ are true hazard rates before and after the change point in the test group. $\lambda_{C}$ is true hazard rate in the control group. T, C, and P represent the results of the test group, control group, and pooled group tested by sequential likelihood ratio method, respectively. Cox represents the test results of Cox regression. CR means censoring rate.

**Supplementary Table S3.** The predictive power, $\alpha$, and $\beta$ values of different methods under various scenarios when the change point is from the 2nd to the 5th month.

| Change point | Scenario | Un-adjusted | MIID Only | Profile likelihood | | Bayesian Exponential | | NP-Area | | NP-KM | |
| --- | --- | --- | --- | --- | --- | --- | --- | --- | --- | --- | --- |
|  |  |  |  | AHR | IWHR | AHR | IWHR | AHR | IWHR | AHR | IWHR |
| 2nd month | 1 | PP=77.9  $\alpha$=0.001  $\beta$=0.36 | PP=78.9  $\alpha$=0.001  $\beta$=0.32 | PP=77.5  $\alpha$=0.009  $\beta$=0.34 | PP=75.5  $\alpha$=0.007  $\beta$=0.35 | PP=77.7  $\alpha$=0.006  $\beta$=0.32 | PP=76.4  $\alpha$=0.005  $\beta$=0.33 | PP=80.7  $\alpha$=0.006  $\beta$=0.29 | PP=81.8  $\alpha$=0.010  $\beta$=0.26 | PP=81.1  $\alpha$=0.009  $\beta$=0.28 | PP=83.0  $\alpha$=0.008  $\beta$=0.26 |
|  | 2 | PP=90.7  $\alpha$=0  $\beta$=0.20 | PP=92.0  $\alpha$=0  $\beta$=0.17 | PP=96.5  $\alpha$=0.002  $\beta$=0.07 | PP=98.0  $\alpha$=0.004  $\beta$=0.04 | PP=95.8  $\alpha$=0.002  $\beta$=0.09 | PP=97.2  $\alpha$=0.002  $\beta$=0.06 | PP=96.2  $\alpha$=0.002  $\beta$=0.08 | PP=97.5  $\alpha$=0.003  $\beta$=0.05 | PP=95.9  $\alpha$=0.002  $\beta$=0.08 | PP=97.7  $\alpha$=0.002  $\beta$=0.04 |
|  | 3 | PP=55.9  $\alpha$=0.003  $\beta$=0.47 | PP=58.6  $\alpha$=0.002  $\beta$=0.42 | PP=68.8  $\alpha$=0.019  $\beta$=0.31 | PP=74.3  $\alpha$=0.030  $\beta$=0.26 | PP=65.0  $\alpha$=0.008  $\beta$=0.35 | PP=70.5  $\alpha$=0.026  $\beta$=0.30 | PP=68.7  $\alpha$=0.015  $\beta$=0.32 | PP=74.4  $\alpha$=0.025  $\beta$=0.27 | PP=68.6  $\alpha$=0.015  $\beta$=0.32 | PP=73.7  $\alpha$=0.021  $\beta$=0.27 |
|  | 4 | PP=6.5  $\alpha$=0.001  $\beta$=0.06 | PP=4.4  $\alpha$=0.001  $\beta$=0.06 | PP=6.3  $\alpha$=0.008  $\beta$=0.06 | PP=7.1  $\alpha$=0.010  $\beta$=0.06 | PP=5.4  $\alpha$=0.003  $\beta$=0.06 | PP=6.6  $\alpha$=0.008  $\beta$=0.06 | PP=6.3  $\alpha$=0.006  $\beta$=0.06 | PP=8.5  $\alpha$=0.012  $\beta$=0.06 | PP=7.3  $\alpha$=0.009  $\beta$=0.06 | PP=8.2  $\alpha$=0.010  $\beta$=0.06 |
|  | 5 | PP=78.6  $\alpha$=0.001  $\beta$=0.32 | PP=85.9  $\alpha$=0.007  $\beta$=0.19 | PP=78.7  $\alpha$=0.006  $\beta$=0.31 | PP=79.2  $\alpha$=0.011  $\beta$=0.30 | PP=76.7  $\alpha$=0.006  $\beta$=0.34 | PP=76.5  $\alpha$=0.006  $\beta$=0.34 | PP=80.0  $\alpha$=0.002  $\beta$=0.28 | PP=80.7  $\alpha$=0.008  $\beta$=0.27 | PP=80.6  $\alpha$=0.004  $\beta$=0.27 | PP=81.6  $\alpha$=0.009  $\beta$=0.26 |
| 3rd month | 1 | PP=77.5  $\alpha$=0.002  $\beta$=0.35 | PP=79.6  $\alpha$=0  $\beta$=0.33 | PP=77.9  $\alpha$=0.006  $\beta$=0.33 | PP=75.9  $\alpha$=0.005  $\beta$=0.33 | PP=78.2  $\alpha$=0.005  $\beta$=0.32 | PP=77.0  $\alpha$=0.003  $\beta$=0.33 | PP=81.5  $\alpha$=0.005  $\beta$=0.29 | PP=82.4  $\alpha$=0.006  $\beta$=0.27 | PP=82.4  $\alpha$=0.007  $\beta$=0.27 | PP=83.6  $\alpha$=0.006  $\beta$=0.25 |
|  | 2 | PP=80.9  $\alpha$=0  $\beta$=0.33 | PP=83.8  $\alpha$=0  $\beta$=0.29 | PP=93.9  $\alpha$=0.003  $\beta$=0.11 | PP=95.9  $\alpha$=0.005  $\beta$=0.08 | PP=92.6  $\alpha$=0.003  $\beta$=0.15 | PP=94.5  $\alpha$=0.002  $\beta$=0.10 | PP=93.0  $\alpha$=0.003  $\beta$=0.13 | PP=95.4  $\alpha$=0.005  $\beta$=0.09 | PP=92.6  $\alpha$=0.003  $\beta$=0.14 | PP=95.0  $\alpha$=0.003  $\beta$=0.1 |
|  | 3 | PP=45.5  $\alpha$=0.002  $\beta$=0.48 | PP=49.3  $\alpha$=0.001  $\beta$=0.43 | PP=65.4  $\alpha$=0.021  $\beta$=0.29 | PP=70.3  $\alpha$=0.042  $\beta$=0.26 | PP=60.5  $\alpha$=0.011  $\beta$=0.31 | PP=66.3  $\alpha$=0.029  $\beta$=0.28 | PP=62.8  $\alpha$=0.013  $\beta$=0.30 | PP=69.7  $\alpha$=0.035  $\beta$=0.25 | PP=64.0  $\alpha$=0.019  $\beta$=0.3 | PP=68.2  $\alpha$=0.037  $\beta$=0.27 |
|  | 4 | PP=6.5  $\alpha$=0.002  $\beta$=0.06 | PP=4.4  $\alpha$=0.001  $\beta$=0.06 | PP=6.7  $\alpha$=0.008  $\beta$=0.06 | PP=7.1  $\alpha$=0.009  $\beta$=0.06 | PP=5.5  $\alpha$=0.006  $\beta$=0.06 | PP=6.5  $\alpha$=0.011  $\beta$=0.06 | PP=6.6  $\alpha$=0.008  $\beta$=0.06 | PP=8.5  $\alpha$=0.012  $\beta$=0.06 | PP=7.6  $\alpha$=0.009  $\beta$=0.06 | PP=8.2  $\alpha$=0.011  $\beta$=0.06 |
|  | 5 | PP=79.3  $\alpha$=0.001  $\beta$=0.30 | PP=85.4  $\alpha$=0.005  $\beta$=0.20 | PP=79.8  $\alpha$=0.012  $\beta$=0.29 | PP=80.0  $\alpha$=0.009  $\beta$=0.28 | PP=77.3  $\alpha$=0.006  $\beta$=0.33 | PP=77.1  $\alpha$=0.005  $\beta$=0.32 | PP=80.1  $\alpha$=0.005  $\beta$=0.28 | PP=81.0  $\alpha$=0.010  $\beta$=0.27 | PP=81.1  $\alpha$=0.011  $\beta$=0.27 | PP=82.2  $\alpha$=0.009  $\beta$=0.25 |
| 4th month | 1 | PP=77.9  $\alpha$=0.002  $\beta$=0.34 | PP=78.9  $\alpha$=0.001  $\beta$=0.32 | PP=77.3  $\alpha$=0.006  $\beta$=0.33 | PP=75.7  $\alpha$=0.005  $\beta$=0.35 | PP=77.7  $\alpha$=0.002  $\beta$=0.33 | PP=76.7  $\alpha$=0.006  $\beta$=0.35 | PP=81.1  $\alpha$=0.002  $\beta$=0.29 | PP=81.8  $\alpha$=0.007  $\beta$=0.28 | PP=81.9  $\alpha$=0.006  $\beta$=0.28 | PP=82.7  $\alpha$=0.005  $\beta$=0.26 |
|  | 2 | PP=67.5  $\alpha$=0  $\beta$=0.51 | PP=72.8  $\alpha$=0  $\beta$=0.43 | PP=90.9  $\alpha$=0.005  $\beta$=0.16 | PP=90.9  $\alpha$=0.006  $\beta$=0.15 | PP=89.0  $\alpha$=0.006  $\beta$=0.19 | PP=89.4  $\alpha$=0.003  $\beta$=0.17 | PP=87.6  $\alpha$=0.003  $\beta$=0.21 | PP=90.7  $\alpha$=0.005  $\beta$=0.15 | PP=88.9  $\alpha$=0.003  $\beta$=0.19 | PP=89.4  $\alpha$=0.005  $\beta$=0.17 |
|  | 3 | PP=36.5  $\alpha$=0.002  $\beta$=0.45 | PP=40.0  $\alpha$=0.002  $\beta$=0.41 | PP=59.2  $\alpha$=0.029  $\beta$=0.28 | PP=60.9  $\alpha$=0.034  $\beta$=0.26 | PP=53.8  $\alpha$=0.017  $\beta$=0.31 | PP=58.4  $\alpha$=0.028  $\beta$=0.28 | PP=53.9  $\alpha$=0.017  $\beta$=0.32 | PP=60.9  $\alpha$=0.033  $\beta$=0.27 | PP=57.8  $\alpha$=0.027  $\beta$=0.29 | PP=58.4  $\alpha$=0.034  $\beta$=0.28 |
|  | 4 | PP=6.6  $\alpha$=0  $\beta$=0.06 | PP=4.5  $\alpha$=0  $\beta$=0.06 | PP=6.5  $\alpha$=0.004  $\beta$=0.05 | PP=7.4  $\alpha$=0.008  $\beta$=0.06 | PP=5.3  $\alpha$=0.002  $\beta$=0.06 | PP=6.6  $\alpha$=0.005  $\beta$=0.06 | PP=6.4  $\alpha$=0.003  $\beta$=0.05 | PP=8.3  $\alpha$=0.011  $\beta$=0.06 | PP=7.4  $\alpha$=0.006  $\beta$=0.06 | PP=8.5  $\alpha$=0.011  $\beta$=0.06 |
|  | 5 | PP=79.4  $\alpha$=0.003  $\beta$=0.30 | PP=84.9  $\alpha$=0.007  $\beta$=0.21 | PP=80.4  $\alpha$=0.014  $\beta$=0.30 | PP=80.4  $\alpha$=0.012  $\beta$=0.29 | PP=77.6  $\alpha$=0.010  $\beta$=0.33 | PP=77.3  $\alpha$=0.008  $\beta$=0.33 | PP=80.2  $\alpha$=0.011  $\beta$=0.29 | PP=81.1  $\alpha$=0.012  $\beta$=0.28 | PP=81.6  $\alpha$=0.013  $\beta$=0.27 | PP=82.5  $\alpha$=0.014  $\beta$=0.26 |
| 5th month | 1 | PP=78.6  $\alpha$=0.003  $\beta$=0.35 | PP=79.7  $\alpha$=0.002  $\beta$=0.31 | PP=78.5  $\alpha$=0.010  $\beta$=0.32 | PP=76.7  $\alpha$=0.003  $\beta$=0.34 | PP=78.4  $\alpha$=0.005  $\beta$=0.33 | PP=77.3  $\alpha$=0.002  $\beta$=0.32 | PP=81.6  $\alpha$=0.004  $\beta$=0.27 | PP=82.2  $\alpha$=0.007  $\beta$=0.27 | PP=82.7  $\alpha$=0.009  $\beta$=0.27 | PP=83.5  $\alpha$=0.003  $\beta$=0.26 |
|  | 2 | PP=52.9  $\alpha$=0  $\beta$=0.64 | PP=60.7  $\alpha$=0  $\beta$=0.53 | PP=87.0  $\alpha$=0.017  $\beta$=0.20 | PP=81.2  $\alpha$=0.005  $\beta$=0.25 | PP=84.2  $\alpha$=0.015  $\beta$=0.24 | PP=80.3  $\alpha$=0.004  $\beta$=0.27 | PP=80.5  $\alpha$=0.006  $\beta$=0.27 | PP=82.6  $\alpha$=0.006  $\beta$=0.24 | PP=84.9  $\alpha$=0.017  $\beta$=0.23 | PP=79.7  $\alpha$=0.005  $\beta$=0.27 |
|  | 3 | PP=27.5  $\alpha$=0.001  $\beta$=0.44 | PP=31.7  $\alpha$=0.001  $\beta$=0.41 | PP=52.8  $\alpha$=0.033  $\beta$=0.28 | PP=50.9  $\alpha$=0.031  $\beta$=0.28 | PP=48.6  $\alpha$=0.025  $\beta$=0.31 | PP=50.0  $\alpha$=0.027  $\beta$=0.30 | PP=46.3  $\alpha$=0.014  $\beta$=0.32 | PP=52.6  $\alpha$=0.031  $\beta$=0.28 | PP=52.0  $\alpha$=0.033  $\beta$=0.29 | PP=49.5  $\alpha$=0.031  $\beta$=0.31 |
|  | 4 | PP=5.9  $\alpha$=0  $\beta$=0.05 | PP=4.0  $\alpha$=0  $\beta$=0.05 | PP=6.2  $\alpha$=0.005  $\beta$=0.05 | PP=6.6  $\alpha$=0.009  $\beta$=0.06 | PP=5.2  $\alpha$=0.002  $\beta$=0.05 | PP=6.7  $\alpha$=0.006  $\beta$=0.05 | PP=6.1  $\alpha$=0.003  $\beta$=0.05 | PP=7.8  $\alpha$=0.007  $\beta$=0.05 | PP=7.0  $\alpha$=0.005  $\beta$=0.05 | PP=7.7  $\alpha$=0.010  $\beta$=0.06 |
|  | 5 | PP=80.2  $\alpha$=0.001  $\beta$=0.30 | PP=84.8  $\alpha$=0.004  $\beta$=0.20 | PP=82.3  $\alpha$=0.011  $\beta$=0.27 | PP=81.4  $\alpha$=0.006  $\beta$=0.26 | PP=78.0  $\alpha$=0.003  $\beta$=0.33 | PP=78.9  $\alpha$=0.003  $\beta$=0.32 | PP=81.9  $\alpha$=0.006  $\beta$=0.28 | PP=82.2  $\alpha$=0.005  $\beta$=0.26 | PP=83.0  $\alpha$=0.009  $\beta$=0.26 | PP=83.3  $\alpha$=0.007  $\beta$=0.24 |

Note: MIID denotes maximum interim information design. PP denotes predictive power (%). AHR1 is the average HR proposed by Kalbfleisch and Prentice. TEHR is time- and event-weighted HR. NP is non-parametric. KM is Kaplan-Meier.

**Supplementary Table S4.** The conditional power, $\alpha$, and $\beta$ values of different methods under various scenarios when the change point is from the 2nd to the 5th month.

| Change point | Scenario | Un-adjusted | MIID Only | Profile likelihood | | Bayesian Exponential | | NP-Area | | NP-KM | |
| --- | --- | --- | --- | --- | --- | --- | --- | --- | --- | --- | --- |
|  |  |  |  | AHR | IWHR | AHR | IWHR | AHR | IWHR | AHR | IWHR |
| 2nd month | 1 | CP=79.0  $\alpha$=0.005  $\beta$=0.31 | CP=79.7  $\alpha$=0.004  $\beta$=0.29 | CP=78.2  $\alpha$=0.011  $\beta$=0.31 | CP=76.0  $\alpha$=0.008  $\beta$=0.33 | CP=78.4  $\alpha$=0.007  $\beta$=0.28 | CP=77.0  $\alpha$=0.008  $\beta$=0.29 | CP=81.5  $\alpha$=0.011  $\beta$=0.25 | CP=82.5  $\alpha$=0.013  $\beta$=0.24 | CP=81.8  $\alpha$=0.014  $\beta$=0.25 | CP=83.8  $\alpha$=0.011  $\beta$=0.22 |
|  | 2 | CP=91.5  $\alpha$=0  $\beta$=0.17 | CP=92.7  $\alpha$=0  $\beta$=0.15 | CP=96.9  $\alpha$=0.002  $\beta$=0.06 | CP=98.3  $\alpha$=0.004  $\beta$=0.03 | CP=96.4  $\alpha$=0.002  $\beta$=0.07 | CP=97.6  $\alpha$=0.002  $\beta$=0.05 | CP=96.7  $\alpha$=0.002  $\beta$=0.06 | CP=97.8  $\alpha$=0.004  $\beta$=0.04 | CP=96.4  $\alpha$=0.002  $\beta$=0.07 | CP=98.0  $\alpha$=0.003  $\beta$=0.04 |
|  | 3 | CP=56.1  $\alpha$=0.004  $\beta$=0.44 | CP=58.8  $\alpha$=0.004  $\beta$=0.40 | CP=69.2  $\alpha$=0.023  $\beta$=0.29 | CP=74.7  $\alpha$=0.036  $\beta$=0.25 | CP=65.4  $\alpha$=0.016  $\beta$=0.32 | CP=70.9  $\alpha$=0.030  $\beta$=0.28 | CP=69.2  $\alpha$=0.018  $\beta$=0.30 | CP=74.9  $\alpha$=0.033  $\beta$=0.25 | CP=69.0  $\alpha$=0.020  $\beta$=0.30 | CP=74.2  $\alpha$=0.027  $\beta$=0.25 |
|  | 4 | CP=5.7  $\alpha$=0.003  $\beta$=0.06 | CP=4.2  $\alpha$=0.001  $\beta$=0.06 | CP=6.0  $\alpha$=0.008  $\beta$=0.06 | CP=6.9  $\alpha$=0.011  $\beta$=0.06 | CP=5.1  $\alpha$=0.004  $\beta$=0.06 | CP=6.3  $\alpha$=0.013  $\beta$=0.07 | CP=6.0  $\alpha$=0.006  $\beta$=0.06 | CP=8.2  $\alpha$=0.013  $\beta$=0.06 | CP=7.0  $\alpha$=0.009  $\beta$=0.06 | CP=8.0  $\alpha$=0.011  $\beta$=0.06 |
|  | 5 | CP=79.6  $\alpha$=0.006  $\beta$=0.27 | CP=87.0  $\alpha$=0.013  $\beta$=0.16 | CP=79.6  $\alpha$=0.011  $\beta$=0.27 | CP=80.0  $\alpha$=0.015  $\beta$=0.25 | CP=77.6  $\alpha$=0.009  $\beta$=0.29 | CP=77.3  $\alpha$=0.011  $\beta$=0.30 | CP=81.0  $\alpha$=0.007  $\beta$=0.24 | CP=81.6  $\alpha$=0.010  $\beta$=0.22 | CP=81.6  $\alpha$=0.008  $\beta$=0.23 | CP=82.6  $\alpha$=0.012  $\beta$=0.22 |
| 3rd month | 1 | CP=78.5  $\alpha$=0.002  $\beta$=0.30 | CP=80.5  $\alpha$=0.001  $\beta$=0.28 | CP=78.5  $\alpha$=0.007  $\beta$=0.29 | CP=76.4  $\alpha$=0.008  $\beta$=0.31 | CP=78.9  $\alpha$=0.006  $\beta$=0.29 | CP=77.7  $\alpha$=0.005  $\beta$=0.31 | CP=82.3  $\alpha$=0.006  $\beta$=0.24 | CP=83.1  $\alpha$=0.009  $\beta$=0.25 | CP=83.2  $\alpha$=0.010  $\beta$=0.23 | CP=84.4  $\alpha$=0.009  $\beta$=0.22 |
|  | 2 | CP=81.7  $\alpha$=0  $\beta$=0.29 | CP=84.6  $\alpha$=0  $\beta$=0.25 | CP=94.5  $\alpha$=0.004  $\beta$=0.10 | CP=96.4  $\alpha$=0.007  $\beta$=0.07 | CP=93.2  $\alpha$=0.003  $\beta$=0.13 | CP=95.0  $\alpha$=0.005  $\beta$=0.09 | CP=93.6  $\alpha$=0.003  $\beta$=0.12 | CP=95.9  $\alpha$=0.006  $\beta$=0.07 | CP=93.2  $\alpha$=0.003  $\beta$=0.12 | CP=95.5  $\alpha$=0.004  $\beta$=0.08 |
|  | 3 | CP=45.3  $\alpha$=0.003  $\beta$=0.45 | CP=49.3  $\alpha$=0.001  $\beta$=0.41 | CP=65.8  $\alpha$=0.030  $\beta$=0.27 | CP=70.7  $\alpha$=0.054  $\beta$=0.25 | CP=60.8  $\alpha$=0.019  $\beta$=0.30 | CP=66.7  $\alpha$=0.040  $\beta$=0.26 | CP=63.2  $\alpha$=0.018  $\beta$=0.28 | CP=70.1  $\alpha$=0.041  $\beta$=0.24 | CP=64.4  $\alpha$=0.026  $\beta$=0.28 | CP=68.6  $\alpha$=0.043  $\beta$=0.25 |
|  | 4 | CP=5.9  $\alpha$=0.003  $\beta$=0.06 | CP=4.2  $\alpha$=0.003  $\beta$=0.06 | CP=6.5  $\alpha$=0.008  $\beta$=0.06 | CP=6.8  $\alpha$=0.010  $\beta$=0.06 | CP=5.2  $\alpha$=0.006  $\beta$=0.06 | CP=6.2  $\alpha$=0.012  $\beta$=0.06 | CP=6.4  $\alpha$=0.008  $\beta$=0.06 | CP=8.2  $\alpha$=0.014  $\beta$=0.06 | CP=7.3  $\alpha$=0.009  $\beta$=0.06 | CP=7.9  $\alpha$=0.012  $\beta$=0.06 |
|  | 5 | CP=80.4  $\alpha$=0.002  $\beta$=0.26 | CP=86.4  $\alpha$=0.013  $\beta$=0.17 | CP=80.6  $\alpha$=0.014  $\beta$=0.25 | CP=80.9  $\alpha$=0.010  $\beta$=0.25 | CP=78.2  $\alpha$=0.009  $\beta$=0.28 | CP=77.9  $\alpha$=0.006  $\beta$=0.28 | CP=81.0  $\alpha$=0.007  $\beta$=0.24 | CP=81.9  $\alpha$=0.013  $\beta$=0.24 | CP=82.0  $\alpha$=0.012  $\beta$=0.23 | CP=83.1  $\alpha$=0.010  $\beta$=0.21 |
| 4th month | 1 | CP=78.8  $\alpha$=0.002  $\beta$=0.29 | CP=79.6  $\alpha$=0.001  $\beta$=0.28 | CP=77.9  $\alpha$=0.009  $\beta$=0.29 | CP=76.2  $\alpha$=0.007  $\beta$=0.31 | CP=78.3  $\alpha$=0.005  $\beta$=0.30 | CP=77.3  $\alpha$=0.007  $\beta$=0.31 | CP=81.9  $\alpha$=0.004  $\beta$=0.25 | CP=82.5  $\alpha$=0.010  $\beta$=0.25 | CP=82.6  $\alpha$=0.010  $\beta$=0.24 | CP=83.5  $\alpha$=0.008  $\beta$=0.24 |
|  | 2 | CP=68.1  $\alpha$=0  $\beta$=0.46 | CP=73.5  $\alpha$=0  $\beta$=0.38 | CP=91.5  $\alpha$=0.008  $\beta$=0.13 | CP=91.4  $\alpha$=0.009  $\beta$=0.13 | CP=89.7  $\alpha$=0.008  $\beta$=0.17 | CP=90.1  $\alpha$=0.006  $\beta$=0.16 | CP=88.3  $\alpha$=0.004  $\beta$=0.18 | CP=91.2  $\alpha$=0.008  $\beta$=0.13 | CP=89.6  $\alpha$=0.006  $\beta$=0.16 | CP=90.0  $\alpha$=0.009  $\beta$=0.15 |
|  | 3 | CP=36.0  $\alpha$=0.002  $\beta$=0.42 | CP=39.8  $\alpha$=0.002  $\beta$=0.39 | CP=59.4  $\alpha$=0.036  $\beta$=0.26 | CP=61.0  $\alpha$=0.049  $\beta$=0.25 | CP=53.8  $\alpha$=0.023  $\beta$=0.29 | CP=58.6  $\alpha$=0.034  $\beta$=0.27 | CP=53.9  $\alpha$=0.022  $\beta$=0.29 | CP=61.1  $\alpha$=0.044  $\beta$=0.26 | CP=58.0  $\alpha$=0.032  $\beta$=0.27 | CP=58.5  $\alpha$=0.044  $\beta$=0.27 |
|  | 4 | CP=5.8  $\alpha$=0.002  $\beta$=0.06 | CP=4.2  $\alpha$=0.001  $\beta$=0.06 | CP=6.2  $\alpha$=0.004  $\beta$=0.05 | CP=7.1  $\alpha$=0.009  $\beta$=0.06 | CP=5.1  $\alpha$=0.002  $\beta$=0.06 | CP=6.3  $\alpha$=0.008  $\beta$=0.06 | CP=6.2  $\alpha$=0.003  $\beta$=0.05 | CP=8.1  $\alpha$=0.012  $\beta$=0.05 | CP=7.1  $\alpha$=0.006  $\beta$=0.05 | CP=8.2  $\alpha$=0.011  $\beta$=0.06 |
|  | 5 | CP=80.3  $\alpha$=0.005  $\beta$=0.25 | CP=85.8  $\alpha$=0.010  $\beta$=0.18 | CP=81.2  $\alpha$=0.019  $\beta$=0.27 | CP=81.2  $\alpha$=0.014  $\beta$=0.25 | CP=78.4  $\alpha$=0.012  $\beta$=0.29 | CP=78.1  $\alpha$=0.010  $\beta$=0.28 | CP=81.1  $\alpha$=0.014  $\beta$=0.27 | CP=81.9  $\alpha$=0.013  $\beta$=0.24 | CP=82.5  $\alpha$=0.019  $\beta$=0.24 | CP=83.4  $\alpha$=0.017  $\beta$=0.23 |
| 5th month | 1 | CP=79.6  $\alpha$=0.005  $\beta$=0.29 | CP=80.5  $\alpha$=0.004  $\beta$=0.27 | CP=79.2  $\alpha$=0.011  $\beta$=0.28 | CP=77.3  $\alpha$=0.006  $\beta$=0.30 | CP=79.1  $\alpha$=0.006  $\beta$=0.29 | CP=77.9  $\alpha$=0.002  $\beta$=0.29 | CP=82.3  $\alpha$=0.007  $\beta$=0.25 | CP=82.8  $\alpha$=0.010  $\beta$=0.23 | CP=83.5  $\alpha$=0.012  $\beta$=0.24 | CP=84.3  $\alpha$=0.006  $\beta$=0.21 |
|  | 2 | CP=53.1  $\alpha$=0  $\beta$=0.58 | CP=61.1  $\alpha$=0  $\beta$=0.48 | CP=87.7  $\alpha$=0.020  $\beta$=0.17 | CP=81.8  $\alpha$=0.005  $\beta$=0.22 | CP=84.8  $\alpha$=0.017  $\beta$=0.20 | CP=80.9  $\alpha$=0.004  $\beta$=0.24 | CP=81.2  $\alpha$=0.006  $\beta$=0.24 | CP=83.2  $\alpha$=0.008  $\beta$=0.21 | CP=85.6  $\alpha$=0.019  $\beta$=0.20 | CP=80.2  $\alpha$=0.006  $\beta$=0.24 |
|  | 3 | CP=26.6  $\alpha$=0.002  $\beta$=0.43 | CP=31.3  $\alpha$=0.001  $\beta$=0.40 | CP=52.9  $\alpha$=0.042  $\beta$=0.27 | CP=50.9  $\alpha$=0.036  $\beta$=0.27 | CP=48.6  $\alpha$=0.031  $\beta$=0.29 | CP=50.0  $\alpha$=0.030  $\beta$=0.28 | CP=46.2  $\alpha$=0.017  $\beta$=0.30 | CP=52.7  $\alpha$=0.037  $\beta$=0.27 | CP=52.0  $\alpha$=0.042  $\beta$=0.28 | CP=49.4  $\alpha$=0.034  $\beta$=0.29 |
|  | 4 | CP=5.1  $\alpha$=0  $\beta$=0.05 | CP=3.8  $\alpha$=0  $\beta$=0.05 | CP=6.0  $\alpha$=0.007  $\beta$=0.05 | CP=6.3  $\alpha$=0.012  $\beta$=0.06 | CP=5.0  $\alpha$=0.005  $\beta$=0.05 | CP=6.4  $\alpha$=0.008  $\beta$=0.05 | CP=5.8  $\alpha$=0.004  $\beta$=0.05 | CP=7.6  $\alpha$=0.012  $\beta$=0.06 | CP=6.7  $\alpha$=0.008  $\beta$=0.05 | CP=7.4  $\alpha$=0.013  $\beta$=0.06 |
|  | 5 | CP=81.1  $\alpha$=0.001  $\beta$=0.24 | CP=85.7  $\alpha$=0.005  $\beta$=0.16 | CP=83.2  $\alpha$=0.015  $\beta$=0.23 | CP=82.3  $\alpha$=0.008  $\beta$=0.23 | CP=78.8  $\alpha$=0.006  $\beta$=0.28 | CP=79.8  $\alpha$=0.004  $\beta$=0.27 | CP=82.9  $\alpha$=0.010  $\beta$=0.23 | CP=83.1  $\alpha$=0.009  $\beta$=0.22 | CP=83.9  $\alpha$=0.013  $\beta$=0.22 | CP=84.2  $\alpha$=0.011  $\beta$=0.21 |

Note: MIID denotes maximum interim information design. CP denotes conditional power (%). AHR1 is the average HR proposed by Kalbfleisch and Prentice. TEHR is time- and event-weighted HR. NP is non-parametric. KM is Kaplan-Meier.

**Supplementary Table S5.** Average number of interim analysis events for unadjusted and maximum interim information design under various scenarios.

| Scenario | Average number for unadjusted method | Average number for MIID with different change points | | | |
| --- | --- | --- | --- | --- | --- |
|  |  | 2nd month | 3rd month | 4th month | 5th month |
| 1 | 174 | 185.3 | 185.1 | 185.5 | 185.4 |
| 2 |  | 179.2 | 181.5 | 184.1 | 186.9 |
| 3 |  | 188.8 | 190.4 | 192.5 | 194.3 |
| 4 |  | 206.5 | 206.4 | 206.4 | 206.6 |
| 5 |  | 174.3 | 174.6 | 175.0 | 175.4 |

Note: MIID denotes maximum interim information design.

**Supplementary Table S6.** Average interim analysis months for the maximum interim information design under various scenarios.

| Scenario | Average months of MIID with different change point | | | |
| --- | --- | --- | --- | --- |
|  | 2nd month | 3rd month | 4th month | 5th month |
| 1 | 18.64 | 18.64 | 18.63 | 18.62 |
| 2 | 19.51 | 19.08 | 18.63 | 18.23 |
| 3 | 18.18 | 17.94 | 17.69 | 17.47 |
| 4 | 16.53 | 16.51 | 16.50 | 16.49 |
| 5 | 21.95 | 21.61 | 21.25 | 20.91 |

Note: MIID denotes maximum interim information design.
